# Supplementary material for: Cas9-specific immune responses compromise local and systemic AAV CRISPR therapy in multiple dystrophic canine models
Source: Nat Commun. 2021 Nov 24;12:6769. doi: 10.1038/s41467-021-26830-7 (PMC8613397; doi:10.1038/s41467-021-26830-7)
Supplement: Supplementary file 1 — Supplementary information [file 41467_2021_26830_MOESM1_ESM.pdf]

Supplementary information

Cas9-specific immune responses compromise local and systemic AAV CRISPR therapy in multiple dystrophic canine models

Chady H. Hakim[1, 2], Sandeep R.P. Kumar[3, 4†], Dennis O. Pérez-López[1 †], Nalinda B. Wasala[1], Dong Zhang[5, 6, 7], Yongping Yue[1], James Teixeira[1], Xiufang Pan[1], Keqing Zhang[1], Emily D. Million[1], Christopher E. Nelson[8, 9], Samantha Metzger[1], Jin Han[1], Jacqueline A. Louderman[1], Florian Schmidt[10, 11, 12], Feng Feng[1], Dirk Grimm[10, 11, 12], Bruce F. Smith[13, 14], Gang Yao[15], N. Nora Yang[2], Charles A. Gersbach[8, 9, 16], Shi-jie Chen[5, 6, 7], Roland W. Herzog[3, 4], Dongsheng Duan[1, 15, 17, 18*]

[1], Department of Molecular Microbiology and Immunology, The University of Missouri, Columbia, MO, USA

[2], National Center for Advancing Translational Sciences, NIH, Rockville, MD, USA

[3], Department of Pediatrics, Indiana University, Indianapolis, IN, USA

[4], Herman B Wells Center for Pediatric Research, Indiana University, Indianapolis, IN, USA

[5], Department of Physics, The University of Missouri, Columbia, MO, USA

[6], Department of Biochemistry, The University of Missouri, Columbia, MO, USA

[7], Institute for Data Science and Informatics, The University of Missouri, Columbia, MO, USA

[8], Department of Biomedical Engineering, Duke University, Durham, NC, USA

[9], Center for Advanced Genomic Technologies Biology, Duke University, Durham, NC, USA

[10], Department of Infectious Diseases/Virology, University of Heidelberg, Heidelberg, Germany

[11], Cluster of Excellence CellNetworks, University of Heidelberg, Heidelberg, Germany

[12], BioQuant, University of Heidelberg, Heidelberg, Germany

[13], Department of Pathobiology, Auburn University, AL, USA

[14], Scott-Ritchey Research Center, Auburn University, AL, USA

[15], Department of Biomedical, Biological & Chemical Engineering, The University of Missouri, Columbia, MO, USA

[16], Department of Surgery, Duke University Medical Center, Durham, NC, USA

[17], Department of Neurology, The University of Missouri, Columbia, MO, USA

[18], Department of Biomedical Sciences, The University of Missouri, Columbia, MO, USA

[†], contributed equally to this work

[*], Corresponding Address: Dongsheng Duan Ph.D.
Department of Molecular Microbiology and Immunology
The University of Missouri School of Medicine
Email: duand@missouri.edu

Supplementary Tables

Supplementary Table 1. Candidate SaCas9 gRNAs designed for GRMD editing and in vitro screening results.

gRNA Number	Sequence (5'-3')	Percentage of indel (%)
	GRMD Intron 5	
CCR64	ttctgcctagggcgtgatcc	ND
CCR65	aagtatggcacagtagctaa	ND
CCR66	agtgtacaaaaacatcagac	ND
CCR67	acttaaaaagtttaatgtag	ND
CCR68	agtttaatgtagcagaatct	2.89
CCR69	gtatggtgacacctaccaat	ND
CCR70	agagtagattcctaactaac	ND
CCR71	ggagtccattctagttagtt	2.28
CCR72	tagaatggactccgtcctgg	ND
CCR73	aataatagattctaccagga	3.25
CCR74	gtaataagcggtaggaattt	ND
CCR75	aatcttcagtaataagcggt	2.69
	GRMD Intron 8	
CCR76	atatgaaactttctgtcagc	ND
CCR77	tattattgtacaggatctag	ND
CCR78	acagtaagaacaagccatgt	1.97
CCR79	tagctttccatgccttcagc	5.87
CCR80	taaatatggaactggcttag	1.97
CCR81	atgaaagctgcagtcctttc	ND
CCR82	ctttccaggatcttgacctg	3.05
CCR83	atatgggatgatgctcttca	3.77
CCR84	gcatcatcccatatgaatgc	7.05
CCR85	catatgaatgctggagttct	ND
CCR86	atgctggagttctgtcagtg	3.93
CCR87	caggtaatcagagaaggtat	4.29

Supplementary Table 2. Primers used in the Surveyor assay for GRMD editing.

Intron	GRMD gRNA	Forward primer (5' - 3')	Reverse primer (5' - 3')	Amplicon size (bp)
Intron 5	CCR64-65	TGCACCATGTTTTGGGCATT	ATGGATATGGCAGGAAGGAGTG	960
	CCR66-75	CACAAACGAGCCAGAGTGCT	GGACCCAGCTCAGGAGAATC	1348
Intron 8	CCR76	TTGCCAAGGCCATCAAAAGT	TTAACACATCAGCAGGTTGCC	574
	CCR77-87	TGGCAACCTGCTGATGTGTT	AGCCTGTGTGTAGGCATAGC	820

Supplementary Table 3. Candidate SpCas9 gRNAs designed for targeting the splicer acceptor for WCMD and LRMD editing and *in vitro* screening results.

gRNA	PAM	Strand	Location	On-target activity	Number of off-target	Specificty score	gRNA ID	Cleavage efficiency (Supplementary Fig. 1d)
CAGAATGAAAATCTAAGACC	AGG	-	Intron 19/New exon	14.430	553	2.63E-01	LRMDSA-1 sgRNA	72.0
AGAATGAAAATCTAAGACCT	CAG	-	Intron 19/New exon	3.607	712	5.08E-02	LRMDSA-2 sgRNA	70.0
TCCTCTTTTAAATTTGTCCT	CAG	+	Intron 19	3.655	902	3.40E-02	LRMDSA-3 sgRNA	67.0
GCCACGATAGCCAAACTGTG	TGG	-	Intron 13/New exon	14.058	274	5.32E-01	WCMDSA-1 sgRNA	71.0
AGGTTACTGCTTTAAATTTC	CAG	+	Intron 13	3.593	499	2.51E-02	WCMDSA-2 sgRNA	24.8
CGATAGCCAAACTGTGGAAG	AGG	-	New exon	14.392	29722	1.37E-04	WCMDSA-3 sgRNA	87.0
CTTTAAATTTCCAGCCACGA	TAG	+	Intron 13/New exon	3.747	248	1.56E-01	WCMDSA-4 sgRNA	17.5
TAAATTTCCAGCCACGATAG	AAG	-	Intron 13/New exon	3.672	150	1.20E-01	WCMDSA-5 sgRNA	31.0

Supplementary Table 4. In vitro screening of gRNAs designed for targeting exonic splicing enhancer for LRMD editing.

gRNA	PAM	Strand	Position[a]	On-target activity	Number of off-target	Specificty score	gRNA ID	Cleavage efficiency (Supplementary Fig. 1d)
GCCATCTTGCTCCACATCTC	AGG	-	148	14.483	443	5.39E-01	LRMD-ESE-1 gRNA	9.3
ACCTGAGATGTGGAGCAAGA	TGG	+	15	14.651	780	1.43E-01		
TGAGATGTGGAGCAAGATGG	CGG	+	18	14.988	1290	1.18E-01		
AAAATCTAAGACCTGAGATG	TGG	+	5	14.963	701	9.25E-02	LRMD-ESE-2 gRNA	96.5
TTGGTGGAGACAGGTGATTT	GGG	-	111	14.071	7597	1.17E-03		
TCTCACTGTTGCATTCCAGC	TGG	-	23	14.758	2679	1.08E-03	LRMD-ESE-3 gRNA	4.4
TGGTGGAGACAGGTGATTTG	GGG	-	112	14.551	9470	9.21E-04		
GAGCAAGATGGCGGAAGAGT	AGG	+	27	14.417	7849	9.14E-04		
CTAGGTAGTTTGGTGGAGAC	AGG	-	102	14.362	6373	9.04E-04		
TGAAGGTTTTCTAGGTAGTT	TGG	-	92	14.035	6023	8.40E-04		
AGGTTTTCTAGGTAGTTTGG	TGG	-	95	14.463	6910	8.16E-04		
TTTGGTGGAGACAGGTGATT	TGG	-	110	13.932	6548	5.39E-04		
GGATAATTTGAAGGTTTTCT	AGG	-	84	13.954	7105	5.04E-04		
ATCCTGAAAATCTATGAATT	CGG	+	99	13.891	6341	4.54E-04		
AGATTTAAAGAGAGACCAGC	TGG	+	126	14.633	7625	4.51E-04		
AGATTTTCAGGATAATTTGA	AGG	-	75	14.239	8328	3.85E-04		
CTGGTCTCTCTTTAAATCTC	AGG	-	42	14.363	7665	3.50E-04		
GGCCGAATTCATAGATTTTC	AGG	-	63	13.701	6725	3.43E-04		
AGCAAGATGGCGGAAGAGTA	GGG	+	28	14.679	8194	2.52E-04		

a, Position refers to the index of the 5’-end of the gRNA

Supplementary Table 5. In vitro screening of gRNAs designed for targeting exonic splicing enhancer for WCMD editing.

gRNA	PAM	Strand	Position[a]	On-target activity	Number of off-target	Specificty score	gRNA ID	Cleavage efficiency (Supplementary Fig. 1d)
TCAACGTGGATGGAACTGGA	GGG	+	159	15.295	48277	4.67E-05		
TCCACGTTGAAGCAAATGGT	GGG	-	53	15.156	45513	5.75E-05	WCMD-ESE-1 gRNA	4.0
CAGCCACGATAGCCAAACTG	TGG	+	28	15.069	18984	2.03E-03	WCMD-ESE-2 gRNA	88.6
TCATCTTTCGTTGGACACCG	AGG	-	142	15.016	24871	1.32E-04	WCMD-ESE-3 gRNA	13.6
GGTGTCCAACGAAAGATGAA	TGG	+	61	14.983	28354	9.02E-05		
GATGTGGTTTATGTATACAA	TGG	+	92	14.938	32275	6.41E-05		
AAATTTAAAGCAGTAACCTG	AGG	-	195	14.869	702	7.53E-02		
TTCAACGTGGATGGAACTGG	AGG	+	158	14.845	45329	5.30E-05		
ACCCACCATTTGCTTCAACG	TGG	+	145	14.832	30826	6.29E-05		
ATGAATGGATAAAGAAGATG	TGG	+	76	14.786	47808	4.56E-05		
CACGATAGCCAAACTGTGGA	AGG	+	32	14.743	31397	1.48E-04		
ATCCACGTTGAAGCAAATGG	TGG	-	52	14.736	44470	5.92E-05		
TGAAGTAAGTCAATTGGAGA	AGG	+	194	14.615	39155	3.52E-04		
TCCATCCACGTTGAAGCAAA	TGG	-	49	14.516	39033	5.95E-05		
CCAAACTGTGGAAGGAGCCT	CGG	+	40	14.472	26455	5.94E-05		
GCTGAGTGAAGTAAGTCAAT	TGG	+	188	14.459	44107	9.08E-05		
ACCATTTGCTTCAACGTGGA	TGG	+	149	14.431	35434	6.95E-05		
CTTCCACAGTTTGGCTATCG	TGG	-	168	14.392	29722	1.37E-04		
TTTATCCATTCATCTTTCGT	TGG	-	133	14.330	40713	1.50E-04	WCMD-ESE-4 gRNA	8.8
CCGAGGCTCCTTCCACAGTT	TGG	-	159	14.174	26228	6.68E-05		
CACAGTTTGGCTATCGTGGC	TGG	-	172	14.058	274	5.32E-01	WCMD-ESE-5 gRNA	11.2
TGCTTCAACGTGGATGGAAC	TGG	+	155	14.015	35409	5.80E-05		

a, Position refers to the index of the 5'-end of the gRNA

Supplementary Table 6. PCR primers used to amplify LRMD and WCMD DNA templates for *in vitro* screening.

Mutation	Forward primer (5' - 3')	Reverse primer (5' - 3')	Amplicon size (bp)
LRMD	CTCTTGGAGCTGAAGGACAAA	TCTTGATAGAAGCGCGAACTC	741
WCMD	CCCTCATTCAACCTGAGTCATAG	CAGCATAATACCCTCCAGTTCC	704

Supplementary Table 7. Sample size and experimental animals.

Figure	Number of dogs	Genotype	Gender	Age at injection (mo)	Study duration (wks)	Immune suppresion	Method of injection	AAV serotype	Construct	Dose (vg/muscle)	Dose (vg/kg)
Fig. 1b-i<br>Supplementary Fig. 5b	1	A	F	1	6	H	Local	AAV8	CK8.SpCas9<br>LRMD-4	5.0E+12<br>5.0E+12	N/A<br>N/A
Fig. 1j-p<br>Supplementary Figs. 5c and 7b, c	2	A	M	44	3, 6	H	Local	AAV8	CK8.SpCas9<br>WCMD-1<br>RSV.AP	5.0E+12<br>5.0E+12<br>5.0E+11	N/A<br>N/A<br>N/A
Fig. 1q (top panel) and r<br>Supplementary Fig. 7g	3	A	F	11	14	S	Local	AAV8	CK8.SERCA2a	3.0E+13	N/A
Fig. 1q (bottom panel) and r<br>Supplementary Fig. 7f, g	2	A	M	8	6, 12, 24,<br>48, 84	S	Local	AAV8	CMV.μdys	1.0E+12	N/A
Fig. 2a (upper 2 panels), b-e	3	N	M	0.25	1, 6	None	Local	AAV8	CK8.SpCas9<br>RSV.AP	5.0E+12<br>5.0E+11	N/A<br>N/A
Fig. 2a (lower panel), b, e	1	N	M	0.25	6	None	Local	AAV8	RSV.AP	1.0E+12	N/A
Fig. 2f (upper 2 panels), g-j	3	N	M/F	1	2, 6	H	Local	AAV8	CK8.SpCas9<br>RSV.AP	5.0E+12<br>5.0E+11	N/A<br>N/A
Fig. 2f (lower panel), j	1	N	M	1	8	S	Local	AAV8	RSV.AP	1.0E+12	N/A
Fig. 2k (upper 2 panels), i-p<br>Supplementary Figs. 5d and 8a, b	6	N	M	40	3, 6	H	Local	AAV8	CK8.SpCas9	3.0E+13	N/A
Fig. 2k (lower panel), p	1	N	M	12	12	S	Local	AAV8	RSV.AP	1.0E+12	N/A
Fig. 3a-p<br>Supplementary Figs. 10, 11, 12a, b and 13	2	A	F	1	3, 6, 12	H	Systemic	AAV8	CK8.SpCas9<br>LRMD-4	N/A<br>N/A	1.0E+14<br>1.0E+14
Fig. 3o, p<br>Supplementary Fig. 12c	1	A	M/F	2.5	6, 24, 88	S	Systemic	AAV8	CK8.μdys	N/A	1.0E+14
Fig. 4a-p<br>Supplementary Fig. 14	1<br>1	N	F	1	3, 6, 12	H	Systemic	AAV8	CK8.SpCas9<br>RSV.AP<br>CK8.SpCas9<br>RSV.AP	N/A<br>N/A<br>N/A<br>N/A	3.0E+13<br>5.0E+12<br>8.0E+12<br>5.0E+12
Fig. 4m-p	1	N	F	3	6, 52	S	Systemic	AAV8	RSV.AP	N/A	5.0E+13
Supplementary Fig. 4b-c	1<br>1	N<br>A	M<br>M	44<br>44	N/A<br>N/A	None<br>None	N/A<br>N/A	N/A<br>N/A	N/A<br>N/A	N/A<br>N/A	N/A<br>N/A
Supplementary Figs. 4b,c and 5a	3	A	M/F	12, 44	3	H	Local	AAV8<br>AAV8	CK8.SaCas9/CK8.SpCas9<br>GR/LR/WCMD gRNA	5.0E+12<br>5.0E+12	N/A<br>N/A
Supplementary Fig. 6a	60<br>18	N/C<br>A	M/F<br>M/F	9-48	N/A	None	N/A	N/A	N/A	N/A	N/A
Supplementary Fig. 6b	29<br>5	N<br>A	M/F<br>M/F	4-80	N/A	None	N/A	N/A	N/A	N/A	N/A
Supplementary Fig. 6c, d	3	C	F	42, 43, 55	N/A	None	N/A	N/A	N/A	N/A	N/A
Supplementary Fig. 6e	23	N/C/A	M/F	0-7	N/A	None	N/A	N/A	N/A	N/A	N/A
Supplementary Fig. 7a	1	A	M	16	3	H	Local	AAV8	CK8.SaCas9<br>GRMD gRNA	5.0E+12<br>5.0E+12	N/A<br>N/A
Supplementary Fig. 7d	1	A	M	45	3, 5	H	Local	AAV8	CK8.SpCas9<br>LRMD-4	5.0E+12<br>5.0E+12	N/A<br>N/A
Supplementary Fig. 8c	3	N	M	33	6	H	N/A	N/A	N/A	N/A	N/A
Supplementary Fig. 9a	2	N	M	16	6	S	Local	AAV8	CK8.SpCas9<br>CB.SpCas9	2.3E+13<br>1.5E+13	N/A<br>N/A
Supplementary Fig. 9b	1	N	M	12	3,6	S	Local	AAV8	CK8.SaCas9<br>RSV.AP	5.0E+12<br>2.5E+11	N/A<br>N/A
Supplementary Fig. 9c	1	N	M	15	3,6	H	Local	AAV9	CK8.SpCas9<br>RSV.AP	5.0E+12<br>5.0E+11	N/A<br>N/A

A, affected; C, carrier; N, normal; M, male; F, female; H, high dose regemen; S, standard dose regimen; N/A, non applicable; None, no immune suppression.

Supplementary Table 8. Primers and probes for TaqMan qPCR and ddPCR.

Target	Forward primer (5' - 3')	Reverse primer (5' - 3')	Probe (5' - 3')
SpCas9	CCGGCAAGACAATCCTGGAT	GATCAGCTGCATGAAGTTTCTGTTG	AAGCCGTCGGACTTC
GRMD-1 and GRMD-2 gRNA (eGFP)	GAGCGCACCATCTTCTTCAAG	TGTCGCCCTCGAACTTCAC	ACGACGGCAACTACA
LRMD-4 gRNA	TTCGATTTCTTGGCTTTATATATCTTGTGGAA	CCAGCATAGCTCTTAAACCAGAATGA	ACGAAACACCGGTCTTAG
WCMD-1 gRNA	AAGTGTAAAGTTGAGATTTCCTTCAGGTT	GCTCTTAAACGCCACGATAGC	ACTGTGCGGTGTACCC
AP (RSV)	CGCTGCTTCGCGATGT	ACCCTAGTCCCCTCAGATACG	ACGGGCCAGATATACG
IL-2	CTCTCCAGGATGCTCACATTTAAGT	TTCTGCTAGACATTGAAGGTGTGTAAA	ACGCCCAAGAAGGCCAC
IL-6	CTGGAGGGAAAAGATGGATGCTT	TCCACAAGACCGGTAGTGATTCT	CAGGTCTCCTGATTGAAC
IL-8	CAGTGGCCCACATTGTGAAAA	AGGCACACCTCATTTCCATTGAA	CTCAGAAATCATTGTAAAGCT
IL-15	GCAGGACGTGATACTTGATTTGGAA	ATGCACATCACTTTCAGTATACAGAGT	CCATATGTATAGATTGAATAAGATTG
IL-18	TGAGGATATGCCCGATTCTGACT	CCAGACCTCTAGTGAGGCTATCTTT	TACAGATAATGCACCCCATACCAT
INF γ	CGGTGGGTCTCTTTTCGTAGATATT	AATTTGGCTCTGAATGATTGTTTTGTCA	CTCTCCTCTCTCCATTTCT
TNF α	CCAAGTGACAAGCCAGTAGCT	CTGGAGCTGCCCCTCAG	ATGTTGTAGCAAACCC

Supplementary Table 9. Blood results from AAV.SpCas9 injected adult normal dogs harvested at 3 weeks post-injection.

			Dog #1							Dog #2							Dog #3						
Post-inj. (wk)	VDML Reference interverals		Baseline	-0.3*	$0.1^{\#}$	$0.4^{\#}$	$1^{\#}$	$2^{\#}$	$3^{\#}$	Baseline	-0.3*	$0.1^{\#}$	$0.4^{\#}$	$1^{\#}$	$2^{\#}$	$3^{\#}$	Baseline	-0.3*	$0.1^{\#}$	$0.4^{\#}$	$1^{\#}$	$2^{\#}$	$3^{\#}$
WBC	4.08 - 14.60	x10^3/uL	11.9	***15.3***	***16.9***	10.3	11.3	9.8	13.9	9.9	9.9	12.6	7.2	12.0	7.8	7.8	6.4	10.7	7.8	8.2	8.1	7.9	6.5
RBC	5.29 - 8.34	x10^6/uL	7.4	7.0	6.7	6.7	5.9	6.0	5.4	7.4	6.8	6.7	6.6	6.2	5.6	5.8	7.9	7.8	7.3	7.7	7.2	6.6	6.0
Hgb	13.30 - 20.80	g/dL	18.3	17.5	16.6	16.6	14.8	15.1	13.5	17.5	16.2	15.9	16.0	14.7	13.6	14.0	19.0	18.9	17.6	18.5	17.2	15.8	14.3
Hct	37.20 - 56.40	%	52.1	49.8	45.3	47.1	41.5	42.4	39.0	48.2	45.0	43.0	44.1	40.5	37.9	39.7	51.0	50.9	45.6	49.8	46.4	43.5	40.2
MCV	62.50 - 72.90	fL	70.4	70.8	67.9	70.4	69.9	70.4	72.4	65.0	66.3	64.5	66.4	65.7	67.3	68.2	64.3	65.1	62.5	64.5	64.7	65.5	67.3
MCH	22.40 - 26.20	pg	24.7	24.9	24.9	24.8	24.9	25.1	25.0	23.6	23.9	23.8	24.1	23.9	24.2	24.1	24.0	24.2	24.1	24.0	24.0	23.8	24.0
MCHC	34.20 - 37.90	g/dL	35.1	35.1	36.6	35.2	35.7	35.6	34.6	36.3	36.0	37.0	36.3	36.3	35.9	35.3	37.3	37.1	38.6	37.1	37.1	36.3	35.6
Platelet Count	140.00 - 350.00	x10^3/uL	242.0	228.0	242.0	244.0	170.0	207.0	270.0	260.0	274.0	237.0	218.0	223.0	181.0	248.0	179.0	241.0	252.0	258.0	283.0	224.0	209.0
Segmented Neutrophil	2.27 - 10.60	x10^3/uL	ND	***11.9***	***13.0***	7.5	9.3	6.3	11.4	ND	6.7	***11.0***	4.9	10.0	5.9	5.8	ND	6.9	6.3	5.2	6.5	5.5	4.5
Band Neutrophil	0.00 - 0.18	x10^3/uL	ND	0.0	0.0	0.0	0.0	0.0	0.1	ND	0.0	0.0	0.0	0.1	0.0	0.0	ND	0.0	0.0	0.0	0.1	0.0	0.0
Lymphocyte	0.83 - 4.80	x10^3/uL	ND	2.1	2.4	1.5	1.0	2.4	1.7	ND	1.8	1.1	1.2	1.3	1.3	1.1	ND	2.3	0.8	1.6	0.7	1.7	1.4
Monocyte	0.05 - 1.24	x10^3/uL	ND	1.2	***1.5***	***1.3***	1.0	0.6	0.4	ND	1.2	0.5	0.9	0.6	0.6	0.8	ND	***1.3***	0.6	1.1	0.7	0.3	0.2
Eosinophil	0.07 - 1.40	x10^3/uL	ND	***0.0***	***0.0***	0.1	***0.0***	0.4	0.3	ND	0.2	***0.0***	0.2	***0.0***	***0.0***	0.1	ND	0.3	0.1	0.2	0.1	0.3	0.3
Basophil	0.00 - 0.03	x10^3/uL	ND	0.0	0.0	0.0	0.0	0.0	0.0	ND	0.0	0.0	0.0	0.0	0.0	0.0	ND	0.0	0.0	0.0	0.0	0.0	0.0
Glucose	81.00 115.00	mg/dL	***64.0***	92.0	83.0	100.0	98.0	80.0	86.0	84.0	***57.0***	100.0	104.0	96.0	92.0	81.0	91.0	***66.0***	99.0	101.0	95.0	85.0	99.0
Urea Nitrogen	8.00 29.00	mg/dL	14.0	27.0	15.0	16.0	12.0	11.0	9.0	19.0	34.0	23.0	18.0	22.0	20.0	16.0	17.0	27.0	19.0	18.0	18.0	15.0	16.0
Creatinine	0.70 1.40	mg/dL	1.0	0.8	0.7	0.9	***0.5***	0.6	***0.5***	0.8	0.8	0.7	0.7	***0.5***	***0.5***	***0.5***	0.8	0.7	***0.6***	0.7	***0.5***	***0.5***	***0.6***
Sodium	145.00 - 151.00	mEq/L	148.0	143.0	143.0	144.0	144.0	145.0	145.0	146.0	147.0	145.0	145.0	141.0	144.0	144.0	143.0	146.0	144.0	145.0	143.0	143.0	143.0
Potassium	3.50 4.90	mEq/L	4.2	4.2	4.5	4.6	4.0	3.9	4.2	4.3	4.3	4.3	4.6	3.6	4.0	4.6	4.0	4.7	4.5	4.8	4.1	3.9	3.7
Chloride	110.00 117.00	mEq/L	109.0	110.0	***106.0***	***105.0***	***109.0***	***107.0***	***108.0***	111.0	115.0	112.0	111.0	110.0	112.0	109.0	111.0	114.0	***109.0***	***109.0***	***109.0***	***107.0***	111.0
Biocarbonate	17.00 26.00	mEq/L	22.0	***15.0***	17.0	***14.0***	***16.0***	20.0	17.0	19.0	***15.0***	***13.0***	***13.0***	***13.0***	***15.0***	17.0	***15.0***	17.0	***16.0***	***14.0***	***15.0***	18.0	20.0
Anion Gap	12.00 20.00	mEq/L	***21.0***	***22.0***	***25.0***	***29.0***	***23.0***	***22.0***	***24.0***	***21.0***	***22.0***	***24.0***	***25.0***	***22.0***	***21.0***	***23.0***	***21.0***	20.0	***23.0***	***27.0***	***23.0***	***22.0***	16.0
Albumin	2.70 3.70	g/dL	3.3	3.4	3.6	3.5	3.4	3.6	3.5	2.9	3.1	3.2	3.3	3.4	3.5	3.2	3.2	3.2	3.3	3.3	3.5	3.6	3.3
Total Protein	5.40 6.90	g/dL	7.0	6.9	7.0	6.9	6.6	7.0	6.6	6.8	6.6	6.6	6.4	6.3	6.4	6.3	6.9	6.8	6.8	6.7	7.0	6.8	6.1
Globulin	2.40 3.70	g/dL	3.7	3.6	3.5	3.4	3.2	3.4	3.1	***3.9***	3.5	3.4	3.1	2.9	2.9	3.1	3.7	3.7	3.5	3.4	3.5	3.2	2.8
Calcium	9.10 10.80	mg/dL	10.2	9.7	10.0	9.9	9.8	10.2	9.7	9.5	9.5	9.3	9.5	9.5	9.8	9.4	9.5	9.2	9.8	9.7	9.8	9.8	9.6
Phosphorus	2.30 5.00	mg/dL	3.0	3.6	3.4	3.9	3.3	3.0	3.7	3.7	***5.2***	3.7	***5.4***	4.4	4.5	4.3	3.0	4.8	4.0	4.9	4.4	4.4	3.4
Magnesium	1.60 2.20	mg/dL		2.0	2.3	2.1	2.0	2.1	2.0		2.0	2.1	2.1	1.8	1.9	1.9		2.2	2.0	2.0	2.0	1.9	1.6
Cholesterol	131.00 320.00	mg/dL	188.0	174.0	166.0	157.0	146.0	149.0	148.0	***106.0***	***124.0***	***111.0***	***99.0***	***98.0***	***102.0***	***115.0***	***111.0***	***129.0***	***127.0***	***121.0***	***113.0***	***106.0***	***104.0***
Total Bilirubin	0.10 - 0.40	mg/dL	0.2	0.3	0.4	0.4	0.3	0.4	0.3	0.2	0.2	0.2	0.2	0.3	0.3	0.3	0.3	0.3	0.2	0.2	0.3	0.3	0.2
ALT	14.00 - 76.00	U/L	***111.0***	39.0	48.0	47.0	57.0	59.0	43.0	***90.0***	***79.0***	76.0	63.0	50.0	44.0	41.0	46.0	46.0	42.0	38.0	32.0	30.0	36.0
ALP	12.00 98.00	U/L	39.0	30.0	44.0	47.0	57.0	53.0	44.0	32.0	32.0	33.0	26.0	24.0	25.0	28.0	54.0	66.0	69.0	60.0	54.0	50.0	44.0
GGT	0.00 8.00	U/L	5.0	5.0	***11.0***	3.0	<3	4.0	<3	5.0	***13.0***	***11.0***	6.0	5.0	4.0	3.0	3.0	3.0	***12.0***	5.0	3.0	6.0	3.0
CK	40 - 226	U/L	124	83	153	74	161	85	97	200	212	***260***	133	342	144	***292***	***239***	***420***	223	95	204	83	87

VDML,Veterinary Medical Diagnostic Laboratory in the University of Missouri Veterinary Medical Teaching Hospital.
Basline, 5 week before injection.
*, With immune suppression.
#, With immune suppression and AAV injection.
Black-bold-italic font, value above the VDML reference intervals.
Gray-bold-italic font,value below the VDML reference intervals.
ND, No data.

Supplementary Table 10. Blood results from AAV.SpCas9 injected adult normal dogs harvested at 6 weeks post-injection.

			Dog 4										Dog 5										Dog 6									
Post-inj. (wk)	VDML Reference interverals		Baseline	-0.3*	0.1#	0.4#	1#	2#	3#	4#	5#	6†	Baseline	-0.3*	0.1#	0.4#	1#	2#	3#	4#	5#	6†	Baseline	-0.3*	0.1#	0.4#	1#	2#	3#	4#	5#	6†
WBC	4.08 - 14.60	x10^3/uL	10.5	13.2	***18.1***	9.7	12.0	12.4	11.0	8.7	10.7	9.9	12.6	13.2	***17.4***	10.0	***14.7***	***15.0***	12.6	10.1	12.3	8.6	12.5	***16.4***	14.6	12.8	12.9	11.3	10.1	9.0	9.1	7.0
RBC	5.29 - 8.34	x10^6/uL	8.7	8.2	7.6	7.9	7.7	7.8	7.2	6.9	7.4	7.1	***8.6***	7.7	7.4	7.3	7.3	7.4	7.2	7.3	7.6	6.4	7.3	6.6	6.2	6.3	6.3	6.3	6.4	6.2	6.2	5.7
Hgb	13.30 - 20.80	g/dL	***21.0***	19.9	18.4	19.3	18.9	19.3	17.5	16.8	18.0	17.2	19.7	17.7	17.4	17.2	17.0	17.5	17.1	17.2	17.8	14.9	17.5	16.0	15.1	15.4	15.6	15.4	15.5	14.9	15.1	14.0
Hct	37.20 - 56.40	%	***57.4***	54.5	48.2	52.3	51.4	50.5	48.9	46.9	50.7	48.2	54.0	49.0	46.2	47.4	46.6	46.9	46.9	47.3	49.7	41.8	50.3	46.0	41.5	44.3	42.4	43.8	42.4	43.0	44.0	40.4
MCV	62.50 - 72.90	fL	66.1	66.2	63.7	66.3	66.8	65.1	67.6	67.9	68.8	67.9	63.1	63.8	62.8	65.1	63.7	63.8	65.3	65.2	65.6	65.6	68.8	69.3	67.3	69.9	66.9	69.5	66.6	69.9	70.9	70.5
MCH	22.40 - 26.20	pg	24.2	24.2	24.3	24.5	24.5	24.9	24.2	24.3	24.4	24.2	23.0	23.0	23.6	23.6	23.3	23.8	23.8	23.7	23.5	23.4	23.9	24.1	24.5	24.3	24.6	24.4	24.3	24.2	24.3	24.4
MCHC	34.20 - 37.90	g/dL	36.6	36.6	***38.2***	36.9	36.8	***38.2***	35.8	35.8	35.5	35.7	36.5	36.1	37.7	36.3	36.5	37.3	36.5	36.4	35.8	35.6	34.8	34.8	36.4	34.8	36.8	35.2	36.6	34.7	34.3	34.7
Platelet Count	140.00 - 350.00	x10^3/uL	259.0	266.0	271.0	256.0	320.0	313.0	251.0	230.0	276.0	300.0	216.0	285.0	259.0	265.0	262.0	274.0	296.0	300.0	279.0	258.0	258.0	257.0	218.0	228.0	202.0	225.0	189.0	295.0	333.0	276.0
Segmented Neutrophil	2.27 - 10.60	x10^3/uL	ND	8.0	***16.1***	6.3	7.7	6.0	7.4	4.6	7.1	8.0	ND	9.2	***14.8***	7.3	***12.5***	10.2	9.1	6.0	8.0	6.1	ND	***13.3***	***13.4***	***10.7***	9.8	7.4	6.9	5.5	5.2	4.3
Band Neutrophil	0.00 - 0.18	x10^3/uL	ND	0.0	0.0	0.0	0.0	0.0	0.0	0.1	0.0	0.1	ND	0.0	0.0	0.0	0.0	0.0	0.0	***2.8***	0.0	0.1	ND	0.0	0.0	0.0	0.0	0.0	0.0	0.0	0.1	0.0
Lymphocyte	0.83 - 4.80	x10^3/uL	ND	3.7	1.4	1.9	3.1	4.2	1.9	3.0	2.6	0.8	ND	2.6	1.2	1.4	1.3	3.8	2.1	0.5	3.6	1.9	ND	2.1	0.9	1.2	2.2	3.1	2.3	2.6	3.3	1.7
Monocyte	0.05 - 1.24	x10^3/uL	ND	1.1	0.5	***1.4***	1.0	1.1	1.0	0.7	0.2	0.4	ND	1.2	1.2	1.1	0.9	0.8	0.8	0.7	0.4	0.5	ND	1.0	0.3	0.8	0.9	0.8	0.6	0.7	0.3	0.5
Eosinophil	0.07 - 1.40	x10^3/uL	ND	0.4	0.0	0.2	0.2	1.1	0.8	0.3	***0.8***	0.6	ND	0.1	0.2	0.2	0.0	0.3	0.6	0.0	0.4	0.0	ND	0.0	0.0	0.1	0.0	0.1	0.3	0.2	0.3	0.5
Basophil	0.00 - 0.03	x10^3/uL	ND	0.0	0.0	0.0	0.0	0.0	0.0	0.0	0.0	0.0	ND	0.0	0.0	0.0	0.0	0.0	0.0	0.0	0.0	0.0	ND	0.0	0.0	0.0	0.0	0.0	0.0	0.0	0.0	0.0
Glucose	81.00 115.00	mg/dL	82.0	81.0	91.0	98.0	***78.0***	***54.0***	***75.0***	***79.0***	***66.0***	87.0	***66.0***	92.0	101.0	95.0	107.0	84.0	***80.0***	***75.0***	***75.0***	***72.0***	***73.0***	95.0	114.0	108.0	99.0	***64.0***	***72.0***	93.0	99.0	86.0
Urea Nitrogen	8.00 29.00	mg/dL	16.0	24.0	18.0	13.0	21.0	25.0	23.0	9.0	17.0	10.0	17.0	25.0	15.0	14.0	18.0	20.0	21.0	9.0	15.0	10.0	26.0	30.0	21.0	18.0	23.0	21.0	22.0	14.0	20.0	16.0
Creatinine	0.70 1.40	mg/dL	0.8	*0.6*	*0.5*	*0.6*	*0.6*	*0.6*	*0.6*	*0.5*	*0.6*	0.7	0.8	0.7	*0.5*	0.7	*0.6*	*0.6*	*0.6*	*0.6*	0.7	*0.6*	0.8	0.7	*0.6*	*0.6*	*0.6*	*0.6*	0.7	0.8	0.7	0.7
Sodium	145.00 - 151.00	mEq/L	145.0	***144.0***	145.0	145.0	146.0	146.0	***144.0***	146.0	147.0	149.0	146.0	***143.0***	***144.0***	***144.0***	145.0	145.0	***144.0***	***143.0***	146.0	***143.0***	147.0	***144.0***	***142.0***	***143.0***	***144.0***	145.0	145.0	145.0	145.0	145.0
Potassium	3.50 4.90	mEq/L	4.3	4.7	4.1	4.8	3.9	3.8	4.8	3.8	4.4	4.3	4.3	4.3	4.2	4.4	3.8	3.9	4.5	4.2	4.5	4.7	4.4	4.5	4.2	4.6	4.4	4.2	4.5	4.4	4.7	4.5
Chloride	110.00 117.00	mEq/L	***107.0***	***107.0***	***105.0***	***102.0***	***107.0***	***104.0***	***106.0***	***108.0***	***109.0***	112.0	***109.0***	***108.0***	***105.0***	***104.0***	***107.0***	***105.0***	***108.0***	***108.0***	***109.0***	***109.0***	***107.0***	***106.0***	***104.0***	***104.0***	***102.0***	***104.0***	110.0	***108.0***	110.0	110.0
Biocarbonate	17.00 26.00	mEq/L	19.0	18.0	18.0	***15.0***	20.0	24.0	22.0	20.0	25.0	19.0	19.0	18.0	18.0	***15.0***	20.0	22.0	18.0	19.0	24.0	20.0	19.0	19.0	20.0	***15.0***	24.0	23.0	17.0	17.0	21.0	19.0
Anion Gap	12.00 20.00	mEq/L	***24.0***	***24.0***	***26.0***	***32.0***	***23.0***	***22.0***	***21.0***	***22.0***	17.0	***22.0***	***22.0***	***22.0***	***25.0***	***29.0***	***22.0***	***22.0***	***23.0***	20.0	18.0	19.0	***26.0***	***24.0***	***23.0***	***29.0***	***22.0***	***22.0***	***23.0***	***24.0***	19.0	***21.0***
Albumin	2.70 3.70	g/dL	3.5	3.7	3.6	3.6	3.5	3.7	3.2	3.4	3.3	3.2	3.2	3.1	3.3	3.4	3.5	3.7	3.5	3.3	3.1	3.1	3.4	3.5	3.3	3.4	3.5	3.7	3.4	3.6	3.1	3.1
Total Protein	5.40 6.90	g/dL	***7.0***	***7.5***	***7.0***	***7.0***	6.5	6.9	6.2	6.6	6.3	6.3	6.9	6.6	6.8	6.8	6.8	6.9	6.6	6.5	6.1	6.0	6.8	***7.0***	6.4	6.5	6.4	6.8	6.4	6.9	6.1	6.1
Globulin	2.40 3.70	g/dL	3.5	3.9	3.4	3.4	3.0	3.2	3.0	3.2	3.0	3.1	3.7	3.5	3.5	3.4	3.3	3.2	3.1	3.2	3.0	2.9	3.4	3.5	3.1	3.1	2.9	3.1	3.0	3.3	3.0	3.0
Calcium	9.10 10.80	mg/dL	10.0	10.3	9.8	9.9	10.0	10.3	10.1	9.9	10.2	9.6	10.0	9.3	9.6	10.0	10.2	10.2	10.0	9.8	9.8	9.5	10.1	9.9	9.5	9.3	10.3	10.2	9.8	10.1	9.8	*8.6*
Phosphorus	2.30 5.00	mg/dL	2.9	3.4	4.0	4.8	*2.2*	2.3	3.7	3.3	3.1	1.0	3.6	2.6	3.5	4.2	*2.0*	2.4	4.3	3.6	2.8	*1.9*	3.5	3.8	3.3	4.2	3.7	4.0	4.3	4.0	3.5	2.7
Magnesium	1.60 2.20	mg/dL		***2.4***	2.2	2.2	2.1	2.3	2.0	1.9	1.9	1.7		2.0	2.1	1.9	1.9	2.1	2.1	1.8	1.9	1.9		2.2	2.2	2.1	2.0	2.2	2.0	1.9	2.0	1.7
Cholesterol	131.00 320.00	mg/dL	206.0	218.0	191.0	187.0	159.0	170.0	169.0	184.0	202.0	200.0	198.0	158.0	149.0	137.0	*123.0*	135.0	142.0	135.0	139.0	153.0	314.0	267.0	204.0	200.0	162.0	163.0	167.0	200.0	198.0	210.0
Total Bilirubin	0.10 - 0.40	mg/dL	0.2	0.3	0.2	0.4	0.2	0.1	0.1	0.2	0.1	0.3	0.3	0.3	0.4	***0.6***	0.3	0.2	0.1	0.2	0.2	0.3	0.2	0.3	0.2	0.3	0.2	0.2	0.2	0.2	0.2	0.3
ALT	14.00 - 76.00	U/L	41.0	53.0	***119.0***	***95.0***	60.0	41.0	35.0	36.0	34.0	44.0	44.0	47.0	48.0	41.0	37.0	31.0	37.0	30.0	28.0	36.0	53.0	57.0	74.0	***84.0***	***133.0***	***129.0***	69.0	53.0	36.0	47.0
ALP	12.00 98.00	U/L	33.0	41.0	80.0	98.0	91.0	66.0	46.0	43.0	34.0	35.0	23.0	20.0	29.0	33.0	32.0	27.0	27.0	21.0	18.0	15.0	34.0	46.0	48.0	57.0	61.0	40.0	28.0	31.0	26.0	24.0
GGT	0.00 8.00	U/L	5.0	***48.0***	***27.0***	***10.0***	***12.0***	6.0	4.0	5.0	3.0	5.0	4.0	5.0	**19.0**	5.0	6.0	4.0	4.0	5.0	3.0	<3	4.0	3.0	9.0	6.0	**12.0**	4.0	3.0	4.0	<3	<3
CK	40 - 226	U/L	90	78	117	96	85	72	81	72	106	***487***	108	94	152	75	88	76	125	76	98	178	60	***277***	115	114	166	116	74	77	112	115

VDML,Veterinary Medical Diagnostic Laboratory in the University of Missouri Veterinary Medical Teaching Hospital.
Basline, 5 week before injection.
*, With immune suppression.
#, With immune suppression and AAV injection.
†, With AAV injection.
Black-bold-italic font, value above the VDML reference intervals.
Gray-bold-italic font,value below the VDML reference intervals.
ND, No data.

Supplementary Table 11. Blood results from adult normal dogs that received high-dose prednisolone immune suppression but did not receive AAV.SpCas9 injection.

			Dog 7								Dog 8								Dog 9							
Post-inj. (wk)	VDML Reference interverals		Baseline	-0.3*	0.1*	1*	2*	3*	4*	5*	Baseline	-0.3*	0.1*	1*	2*	3*	4*	5*	Baseline	-0.3*	0.1*	1*	2*	3*	4*	5*
WBC	4.08 - 14.60	x10^3/uL	12.8	13.0	11.7	13.8	8.7	9.6	8.5	8.5	9.9	***16.9***	13.6	***15.1***	7.9	7.5	7.7	7.5	9.7	***15.6***	10.5	13.3	8.6	9.5	9.6	6.6
RBC	5.29 - 8.34	x10^6/uL	8.0	6.9	7.3	6.7	6.6	6.6	7.0	6.9	7.4	6.9	7.2	6.7	6.6	6.8	6.8	7.0	7.9	7.0	7.3	7.0	6.6	6.8	7.1	6.7
Hgb	13.30 - 20.80	g/dL	19.4	17.0	18.0	16.4	16.4	16.5	17.5	17.1	17.4	16.5	17.1	16.1	16.0	16.2	16.6	17.0	19.7	17.0	17.9	17.3	16.4	16.9	17.7	16.8
Hct	37.20 - 56.40	%	55.1	49.1	50.1	47.8	45.3	45.0	49.9	48.8	49.2	47.5	47.7	45.6	44.7	43.5	46.7	46.5	53.4	47.9	49.3	47.6	45.7	45.1	48.5	46.1
MCV	62.50 - 72.90	fL	69.1	71.1	68.7	71.4	68.4	67.9	71.2	70.7	66.7	68.9	66.5	67.7	67.3	63.7	68.3	66.5	67.3	68.9	67.7	68.3	69.1	66.5	68.8	68.6
MCH	22.40 - 26.20	pg	24.3	24.6	24.7	24.5	24.8	25.2	25.0	24.8	23.6	23.9	23.8	23.9	24.1	23.7	24.3	24.3	24.8	24.5	24.6	24.8	24.8	24.9	25.1	25.0
MCHC	34.20 - 37.90	g/dL	35.2	34.6	35.9	34.3	36.2	37.1	35.1	35.0	35.4	34.7	35.8	35.3	35.8	37.2	35.5	36.6	36.9	35.5	36.3	36.3	35.9	37.5	36.5	36.4
Platelet Count	140.00 - 350.00	x10^3/uL	291.0	282.0	***401.0***	***449.0***	***371.0***	313.0	***362.0***	270.0	192.0	239.0	252.0	275.0	219.0	265.0	253.0	230.0	251.0	290.0	309.0	***360.0***	287.0	334.0	350.0	281.0
Segmented Neutrophil	2.27 - 10.60	x10^3/uL	ND	10.3	7.9	**11.3**	6.1	7.5	4.7	5.2	ND	***15.5***	***11.1***	***12.5***	4.6	4.7	4.1	3.9	ND	***14.0***	8.5	10.5	6.2	6.9	6.4	3.9
Band Neutrophil	0.00 - 0.18	x10^3/uL	ND	0.0	0.0	0.0	0.0	0.0	0.0	0.0	ND	0.2	0.0	0.0	0.0	0.0	0.0	0.0	ND	***0.8***	0.0	0.0	0.0	0.0	0.0	0.0
Lymphocyte	0.83 - 4.80	x10^3/uL	ND	1.8	2.6	1.7	2.1	1.6	3.1	2.5	ND	0.5	1.4	2.1	2.4	2.4	2.4	2.6	ND	0.8	1.3	2.0	1.9	2.2	1.9	2.2
Monocyte	0.05 - 1.24	x10^3/uL	ND	0.9	1.2	0.8	0.4	0.5	0.6	0.5	ND	0.7	1.1	0.5	0.5	0.3	0.7	0.6	ND	0.0	0.6	0.8	0.4	0.4	0.9	0.3
Eosinophil	0.07 - 1.40	x10^3/uL	ND	0.0	0.0	0.0	0.1	0.0	0.2	0.3	ND	0.0	0.0	0.0	0.4	0.1	0.5	0.4	ND	0.0	0.1	0.0	0.1	0.0	0.4	0.1
Basophil	0.00 - 0.03	x10^3/uL	ND	0.0	0.0	0.0	0.0	0.0	0.0	0.0	ND	0.0	0.0	0.0	0.0	0.0	0.0	0.0	ND	0.0	0.0	0.0	0.0	0.0	0.0	0.0
Glucose	81.00 115.00	mg/dL	110.0	109.0	***121.0***	***132.0***	95.0	97.0	99.0	94.0	***80.0***	116.0	107.0	***121.0***	***69.0***	87.0	82.0	***79.0***	***65.0***	114.0	104.0	105.0	84.0	***72.0***	85.0	95.0
Urea Nitrogen	8.00 29.00	mg/dL	28.0	15.0	24.0	24.0	17.0	15.0	23.0	11.0	27.0	17.0	22.0	25.0	18.0	14.0	20.0	27.0	29.0	17.0	28.0	29.0	18.0	14.0	24.0	13.0
Creatinine	0.70 1.40	mg/dL	0.9	0.6	0.7	0.8	0.7	0.6	0.8	0.7	0.8	0.6	0.7	0.7	0.8	0.6	0.7	0.7	0.9	0.6	0.7	0.7	0.7	0.6	0.7	0.7
Sodium	145.00 - 151.00	mEq/L	148.0	***144.0***	145.0	***144.0***	148.0	***144.0***	***144.0***	146.0	146.0	148.0	***143.0***	***141.0***	146.0	146.0	145.0	144.0	149.0	149.0	145.0	***143.0***	148.0	147.0	145.0	148.0
Potassium	3.50 4.90	mEq/L	4.5	4.1	4.4	3.9	4.5	4.2	3.9	4.2	4.6	4.2	4.6	4.1	4.0	4.1	4.3	4.4	4.6	4.5	4.4	4.2	4.4	3.8	4.3	3.8
Chloride	110.00 117.00	mEq/L	***107.0***	***105.0***	***104.0***	***103.0***	***103.0***	***104.0***	***105.0***	111.0	111.0	***108.0***	***101.0***	***102.0***	***107.0***	***106.0***	***105.0***	***109.0***	112.0	***108.0***	***104.0***	***102.0***	***106.0***	***104.0***	***106.0***	109.0
Biocarbonate	17.00 26.00	mEq/L	20.0	***15.0***	20.0	18.0	21.0	18.0	20.0	16.0	18.0	***15.0***	20.0	19.0	21.0	19.0	20.0	20.0	18.0	***15.0***	19.0	20.0	22.0	17.0	19.0	19.0
Anion Gap	12.00 20.00	mEq/L	***26.0***	***29.0***	***26.0***	***27.0***	***29.0***	***27.0***	***23.0***	***23.0***	***22.0***	***30.0***	***26.0***	***25.0***	***22.0***	***25.0***	***24.0***	20.0	***23.0***	***31.0***	***26.0***	***25.0***	***24.0***	***29.0***	***25.0***	***24.0***
Albumin	2.70 3.70	g/dL	3.5	3.7	3.7	3.6	3.6	***3.8***	3.5	3.4	3.3	3.7	4.0	***3.9***	3.5	4.0	***3.8***	3.5	3.4	3.6	***3.9***	***3.9***	***3.8***	***4.0***	***3.8***	3.6
Total Protein	5.40 6.90	g/dL	6.7	6.7	6.7	6.5	6.5	***7.0***	6.5	6.1	6.3	6.7	6.8	6.5	6.3	***7.0***	6.5	6.2	6.5	6.5	***7.0***	***7.0***	***7.0***	***7.4***	6.8	6.4
Globulin	2.40 3.70	g/dL	3.3	3.1	3.0	2.9	2.9	3.3	3.0	2.7	3.0	3.0	2.8	2.6	2.8	3.0	2.7	2.7	3.1	2.9	3.1	3.1	3.2	3.4	3.1	2.8
Calcium	9.10 10.80	mg/dL	10.4	9.7	9.8	9.8	10.0	10.1	10.1	10.2	9.7	9.5	10.0	9.8	10.3	10.8	10.5	9.9	10.3	9.6	10.6	10.4	***10.9***	10.7	***10.9***	10.7
Phosphorus	2.30 5.00	mg/dL	***2.2***	3.5	3.0	***1.5***	***2.0***	2.9	***1.9***	***5.1***	3.0	3.7	3.8	3.1	3.7	3.7	3.5	3.9	4.4	3.3	3.1	2.8	***2.0***	3.0	2.8	4.2
Magnesium	1.60 2.20	mg/dL		2.2	***2.5***	2.1	2.0	***2.5***	2.0	1.8		2.0	***2.4***	1.9	1.7	2.1	2.0	1.9		2.2	***2.5***	***2.4***	2.1	2.2	2.1	1.8
Cholesterol	131.00 320.00	mg/dL	206.0	174.0	167.0	**130.0**	**112.0**	187.0	155.0	159.0	218.0	224.0	207.0	153.0	131.0	192.0	189.0	176.0	151.0	152.0	167.0	144.0	**130.0**	170.0	181.0	167.0
Total Bilirubin	0.10 - 0.40	mg/dL	0.3	***0.5***	0.2	0.2	0.2	0.4	0.1	0.2	0.2	0.3	0.3	0.2	0.1	0.2	0.1	0.2	0.1	0.3	0.2	0.2	0.1	0.2	0.1	0.1
ALT	14.00 - 76.00	U/L	39.0	40.0	39.0	47.0	44.0	45.0	30.0	49.2	***84.0***	***191.0***	***172.0***	***134.0***	***299.0***	83.0	60.0	41.0	35.0	***101.0***	***89.0***	***80.0***	***143.0***	47.0	34.0	26.0
ALP	12.00 98.00	U/L	28.0	42.0	42.0	37.0	22.0	24.0	23.0	30.0	18.0	51.0	74.0	79.0	49.0	33.0	26.0	14.0	18.0	28.0	34.0	44.0	31.0	25.0	21.0	16.0
GGT	0.00 8.00	U/L	4.0	5.0	7.0	***12.0***	7.0	***8.4***	4.0	***10.0***	4.0	7.0	***12.0***	***11.0***	7.0	7.0	5.0	3.0	3.0	4.0	***11.0***	***11.0***	6.0	6.0	8.0	3.0
CK	40 - 226	U/L	90	67	149	93	189	188	142	176	64	53	111	61	131	87	133	126	111	64	102	85	138	97	167	123

VDML,Veterinary Medical Diagnostic Laboratory in the University of Missouri Veterinary Medical Teaching Hospital.
Basline, 5 week before injection.
*, With immune suppression.
Black-bold-italic font, value above the VDML reference intervals.
Gray-bold-italic font,value below the VDML reference intervals.
ND, No data.

Supplementary Table 12. Blood results from 1-month-old LRMD dogs that received systemic CRISPR therapy.

			Dog #1							Dog #2										
Post-inj. (wk)	Duan Lab colony reference for DMD dogs (Age 4 to 16 weeks)		Baseline	1[#]	2[#]	3[#]	4[#]	5[#]	6[†]	Baseline	1[#]	2[#]	3[#]	4[#]	5[#]	6[†]	7[†]	8[†]	9[†]	12[†]
WBC	7.45 - 24.13	x10^3/uL	15.90	10.46	11.38	16.27	14.66	10.27	10.63	ND	10.05	10.41	15.54	14.10	8.81	9.38	16.10	17.66	17.29	17.77
RBC	3.89 - 6.25	x10^6/uL	4.57	5.13	4.94	4.71	4.68	4.91	4.79	ND	4.78	4.79	4.68	4.27	5.05	5.03	5.46	5.49	5.80	5.73
Hgb	9.20 - 14.60	g/dL	10.90	12.20	11.40	10.80	10.60	10.90	10.30	ND	11.20	11.10	10.80	9.70	11.50	11.50	12.50	12.60	13.30	13.10
Hct	29.00 - 43.00	%	35.40	36.90	34.40	32.50	32.70	33.00	30.80	ND	34.20	33.40	32.40	30.30	35.20	34.10	36.10	37.30	37.40	36.90
MCV	62.00 - 80.00	fL	77.50	71.90	69.60	69.00	69.90	67.20	64.30	ND	71.50	69.70	69.20	71.00	69.70	67.80	66.10	67.90	64.50	64.40
MCH	20.80 - 24.90	pg	23.90	23.80	23.10	22.90	22.60	22.20	21.50	ND	23.40	23.20	23.10	22.70	22.80	22.90	22.90	23.00	22.90	22.90
MCHC	29.80 - 35.50	g/dL	30.80	33.10	33.10	33.20	32.40	33.00	33.40	ND	32.70	33.20	33.30	32.00	32.70	33.70	34.60	33.80	35.60	35.50
Platelet Count	271.00 - 974.00	x10^3/uL	456.00	***1137.00***	***1152.00***	697.00	800.00	739.00	842.00	ND	***1049.00***	***1216.00***	865.00	833.00	908.00	812.00	1148.00	964.00	908.00	1073.00
Segmented Neutrophil	3.51 - 10.55	x10^3/uL	3.50	4.50	6.83	8.30	9.09	5.03	5.21	ND	4.22	4.37	10.10	7.90	3.70	5.72	10.14	***10.60***	9.86	9.06
Band Neutrophil	0.00 - 0.29	x10^3/uL	0.00	0.00	0.00	0.00	0.00	0.00	0.00	ND	0.10	0.00	0.00	0.00	0.00	0.00	0.00	0.00	0.00	0.00
Lymphocyte	1.71 - 10.16	x10^3/uL	***11.77***	5.23	3.75	6.18	5.13	4.83	4.36	ND	4.92	4.37	3.88	5.50	4.23	3.38	4.67	5.65	6.22	8.17
Monocyte	0.00 - 1.30	x10^3/uL	0.16	0.52	0.46	***1.63***	0.29	0.41	0.64	ND	0.20	***1.56***	***1.40***	0.70	0.62	0.28	1.13	***1.41***	1.21	0.35
Eosinophil	0.00 - 0.57	x10^3/uL	0.48	0.21	0.34	0.16	0.15	0.00	0.42	ND	***0.60***	0.10	0.15	0.00	0.26	0.00	0.16	0.00	0.00	0.18
Basophil	0.00 - 0.14	x10^3/uL	0.00	0.00	0.00	0.00	0.00	0.00	0.00	ND	0.00	0.00	0.00	0.00	0.00	0.00	0.00	0.00	0.00	0.00
Glucose	67.00 - 149.00	mg/dL	140.00	146.00	141.00	117.00	108.00	114.00	132.00	ND	141.00	***156.00***	113.00	137.00	120.00	128.00	100.00	110.00	107.00	95.00
Urea Nitrogen	4.00 - 23.00	mg/dL	10.00	10.00	12.00	6.00	16.00	14.00	11.00	ND	10.00	10.00	9.00	20.00	15.00	11.00	20.00	16.00	18.00	14.00
Creatinine	0.20 - 0.80	mg/dL	0.20	0.30	0.30	0.20	0.60	0.60	0.30	ND	0.40	0.30	0.20	0.80	0.60	0.30	0.80	0.60	0.70	0.50
Sodium	137.00 - 147.00	mEq/L	140.00	142.00	142.00	142.00	145.00	143.00	143.00	ND	146.00	143.00	141.00	144.00	143.00	143.00	145.00	147.00	145.00	143.00
Potassium	4.70 - 6.60	mEq/L	6.10	***7.00***	6.20	6.40	5.80	5.90	5.70	ND	6.10	6.50	6.10	5.70	5.60	5.60	5.90	5.00	5.30	5.60
Chloride	102.00 - 108.00	mEq/L	103.00	103.00	101.00	106.00	104.00	105.00	106.00	ND	105.00	100.00	105.00	102.00	104.00	106.00	104.00	104.00	106.00	105.00
Biocarbonate	19.00 - 26.00	mEq/L	24.00	23.00	22.00	19.00	23.00	23.00	21.00	ND	25.00	21.00	19.00	23.00	24.00	22.00	23.00	22.00	19.00	19.00
Anion Gap	15.00 - 26.00	mEq/L	19.00	23.00	25.00	23.00	24.00	21.00	22.00	ND	22.00	***29.00***	23.00	25.00	21.00	21.00	24.00	26.00	25.00	25.00
Albumin	2.10 - 3.40	g/dL	2.40	2.90	3.00	2.70	3.00	3.00	2.90	ND	2.70	3.10	2.80	3.10	3.10	3.00	3.00	3.20	3.10	3.10
Total Protein	4.00 - 6.20	g/dL	4.50	5.20	5.60	5.40	5.30	5.30	5.40	ND	4.90	5.60	5.20	5.40	5.50	5.40	5.30	5.60	5.80	5.70
Globulin	1.60 - 3.70	g/dL	2.10	2.30	2.60	2.70	2.30	2.30	2.50	ND	2.20	2.50	2.40	2.30	2.40	2.40	2.30	2.40	2.70	2.60
Calcium	10.50 - 12.80	mg/dL	11.70	12.30	11.70	11.00	11.80	11.30	11.30	ND	11.70	11.30	11.30	11.30	11.50	11.50	11.30	11.80	11.40	11.90
Phosphorus	6.90 - 11.00	mg/dL	9.40	10.00	9.20	8.80	10.70	9.60	8.70	ND	9.60	8.80	9.40	10.80	9.30	8.20	10.10	9.90	9.90	9.30
Magnesium	1.30 - 1.90	mg/dL	1.80	***2.20***	***2.00***	1.80	1.90	1.80	1.90	ND	***2.20***	***2.00***	1.80	***2.00***	1.90	1.80	1.80	***2.00***	***2.00***	1.80
Cholesterol	169.00 - 392.00	mg/dL	282.00	264.00	283.00	281.00	179.00	208.00	193.00	ND	239.00	276.00	248.00	170.00	220.00	196.00	208.00	221.00	240.00	238.00
Total Bilirubin	0.10 - 0.30	mg/dL	0.10	0.10	0.10	0.10	0.10	0.10	0.10	ND	0.20	0.20	0.20	0.20	0.10	0.20	0.10	0.20	0.20	0.20
ALT	83.00 - 819.00	U/L	240.00	660.00	565.00	619.00	281.00	362.00	466.00	ND	452.00	598.00	584.00	340.00	539.00	579.00	594.00	610.00	591.00	628.00
ALP	58.00 - 301.00	U/L	132.00	107.00	92.00	97.00	108.00	94.00	90.00	ND	123.00	103.00	91.00	98.00	87.00	86.00	98.00	85.00	110.00	76.00
GGT	1.00 - 10.00	U/L	<3	3.00	<3	<3	<3	<3	<3	ND	3.00	<3	<3	<3	<3	<3	<3	<3	<3	<3
CK	4266 - 209625	U/L	19201	94946	46540	31710	19701	35527	58942	ND	21015	21708	35488	25458	28544	47224	75418	48488	32953	51811

Basline: 1 week before injection

#, With immune suppression and AAV injection

†, With AAV injection

Black-bold-italic font, value above the VDML reference intervals.

ND, No data

Supplementary Table 13. Blood results from 1-month-old normal dogs that received systemic AAV.CK8.SpCas9 injection.

Dog #1

Post-inj. (wk)	Duan Lab colony reference for normal dogs (Age 4 to 16 weeks)		Baseline	1[#]	2[#]	3[#]	4[#]	5[#]	6[†]	7[†]	8[†]	9[†]	10[†]	11[†]	12[†]
WBC	4.44 - 21.30	x10^3/uL	11.8	11.94	9.28	9.96	9.47	9.56	8.22	11.41	9.81	9.45	10.85	8.59	9.62
RBC	2.87 - 6.52	x10^6/uL	3.9	4.53	4.53	4.69	4.95	5.14	5.35	5.34	5.44	5.41	5.67	5.71	5.62
Hgb	8.30 - 15.30	g/dL	9.4	10.70	10.80	11.00	11.60	12.10	12.30	12.50	12.90	12.90	13.70	13.60	13.60
Hct	25.80 - 44.00	%	31.3	35.10	32.80	33.70	35.40	36.40	37.20	37.60	37.70	37.40	38.10	38.90	36.20
MCV	56.00 - 79.70	fL	79.6	77.50	72.40	71.90	71.50	70.80	69.50	70.40	69.30	69.10	67.20	68.10	64.40
MCH	16.90 - 24.60	pg	23.9	23.60	23.80	23.50	23.40	23.50	23.00	23.40	23.70	23.80	24.20	23.80	24.20
MCHC	29.80 - 38.40	g/dL	30.0	30.50	32.90	32.60	32.80	33.20	33.10	33.20	34.20	34.50	36.00	35.00	37.60
Platelet Count	68.00 - 744.00	x10^3/uL	421.0	***814.00***	474.00	566.00	520.00	602.00	404.00	535.00	543.00	588.00	537.00	457.00	489.00
Segmented Neutrophil	4.19 - 9.48	x10^3/uL	7.3	7.28	7.24	7.07	6.06	5.93	4.36	5.59	5.20	5.58	5.75	4.29	5.48
Band Neutrophil	0.00 - 0.23	x10^3/uL	0.0	0.00	0.00	0.00	0.00	0.00	0.00	0.00	0.00	0.00	0.00	0.00	0.00
Lymphocyte	0.76 - 11.93	x10^3/uL	3.4	3.70	1.49	1.89	2.75	2.77	3.37	4.45	4.32	2.74	4.01	3.61	3.37
Monocyte	0.00 - 2.24	x10^3/uL	0.9	0.84	0.37	0.90	0.57	0.67	0.41	0.57	0.29	0.38	0.76	0.60	0.48
Eosinophil	0.00 - 0.86	x10^3/uL	0.1	0.12	0.19	0.10	0.10	0.19	0.08	0.80	0.00	0.66	0.22	0.09	0.19
Basophil	0.00 - 0.08	x10^3/uL	0.0	0.00	0.00	0.00	0.00	0.00	0.00	0.00	0.00	0.10	0.11	0.00	0.10
Glucose	76.00 - 155.00	mg/dL	122.0	118.00	114.00	122.00	116.00	116.00	115.00	103.00	105.00	111.00	104.00	100.00	99.00
Urea Nitrogen	4.00 - 30.00	mg/dL	12.0	14.00	12.00	7.00	11.00	6.00	11.00	15.00	12.00	9.00	21.00	11.00	12.00
Creatinine	0.10 - 1.10	mg/dL	0.3	0.30	0.30	0.30	0.50	0.50	0.50	0.60	0.50	0.30	0.60	0.40	0.40
Sodium	133.00 - 149.00	mEq/L	141.0	143.00	144.00	141.00	143.00	142.00	144.00	145.00	145.00	141.00	144.00	143.00	145.00
Potassium	4.60 - 7.10	mEq/L	5.3	5.20	4.80	5.00	5.10	5.40	5.30	5.40	5.40	5.50	5.60	5.30	5.10
Chloride	99.00 - 112.00	mEq/L	105.0	100.00	105.00	105.00	105.00	104.00	107.00	106.00	107.00	105.00	107.00	109.00	110.00
Biocarbonate	7.00 - 27.00	mEq/L	20.0	23.00	19.00	18.00	21.00	19.00	19.00	20.00	21.00	18.00	21.00	16.00	15.00
Anion Gap	14.00 - 28.00	mEq/L	21.0	25.00	25.00	23.00	22.00	24.00	23.00	24.00	22.00	24.00	22.00	23.00	25.00
Albumin	2.10 - 3.60	g/dL	2.7	3.00	2.70	2.90	2.60	2.70	2.60	2.60	2.60	3.00	2.80	2.90	2.90
Total Protein	4.10 - 6.30	g/dL	5.0	5.50	5.20	5.30	5.00	5.20	4.80	4.70	4.80	5.30	5.00	4.90	5.20
Globulin	1.60 - 3.00	g/dL	2.3	2.50	2.50	2.40	2.40	2.50	2.20	2.10	2.20	2.30	2.20	2.00	2.30
Calcium	10.30 - 12.10	mg/dL	11.4	11.00	10.60	11.40	10.60	11.00	10.70	10.70	10.90	10.80	11.00	10.70	10.70
Phosphorus	7.10 - 11.10	mg/dL	8.2	8.80	9.80	8.70	8.10	9.60	9.80	8.90	9.60	9.10	8.80	8.60	8.50
Magnesium	1.40 - 2.00	mg/dL	1.8	***2.10***	1.80	1.80	1.80	1.80	1.80	1.70	1.70	1.90	1.70	1.80	1.70
Cholesterol	162.00 - 427.00	mg/dL	316.0	304.00	184.00	223.00	223.00	245.00	219.00	225.00	228.00	241.00	232.00	229.00	219.00
Total Bilirubin	0.09 - 0.40	mg/dL	0.1	0.20	0.10	0.10	0.10	0.10	0.10	0.10	0.10	0.30	0.10	0.10	0.10
ALT	9.00 - 121.00	U/L	16.0	31.00	69.00	71.00	55.00	61.00	91.00	107.00	109.00	***145.00***	***129.00***	97.00	87.00
ALP	103.00 - 231.00	U/L	216.0	***284.00***	***316.00***	***280.00***	***279.00***	***303.00***	***273.00***	***251.00***	***242.00***	225.00	226.00	202.00	220.00
GGT	2.00 - 13.00	U/L	3.0	<3	3.00	<3	<3	3.00	3.00	3.00	3.00	7.00	3.00	5.00	<3
CK	235 - 4518	U/L	592.0	460	942	701	519	790	2474	2361	***5825***	3968	***4686***	1015	598

Dog #2

Post-inj. (wk)	Baseline	1[#]	2[#]	3[#]	4[#]	5[#]	6[†]	7[†]	8[†]	9[†]	10[†]	11[†]	12[†]
WBC	13.4	13.71	12.85	8.75	8.37	9.25	9.18	10.48	9.56	9.07	9.79	7.23	7.73
RBC	4.5	4.60	5.20	4.30	4.95	5.46	5.53	5.34	5.25	5.53	5.53	5.28	5.34
Hgb	10.3	10.60	12.10	10.00	11.50	12.60	13.10	12.40	12.40	13.00	13.20	12.30	12.40
Hct	33.8	33.80	36.60	30.40	34.80	37.90	37.80	36.90	34.90	37.90	37.90	35.10	36.10
MCV	75.4	73.50	70.40	70.70	70.30	69.40	68.40	69.10	66.50	68.50	68.50	66.50	67.60
MCH	23.0	23.00	23.30	23.30	23.20	23.10	23.70	23.20	23.60	23.50	23.90	23.30	23.20
MCHC	30.5	31.40	33.10	32.90	33.00	33.20	34.70	33.60	35.50	34.30	34.80	35.00	34.30
Platelet Count	554.0	744.00	620.00	446.00	594.00	690.00	427.00	605.00	582.00	553.00	586.00	465.00	514.00
Segmented Neutrophil	6.8	8.64	7.84	6.21	5.52	6.38	5.97	5.34	4.88	4.44	5.09	4.12	3.63
Band Neutrophil	0.0	0.00	0.13	0.00	0.00	0.00	0.00	0.00	0.10	0.00	0.00	0.00	0.04
Lymphocyte	5.4	3.29	3.08	1.66	1.67	2.40	2.48	2.93	3.73	3.63	3.82	2.39	3.40
Monocyte	1.1	1.65	1.67	0.70	1.00	0.37	0.73	1.05	0.67	0.64	0.69	0.65	0.39
Eosinophil	0.1	0.14	0.13	0.17	0.17	0.09	0.00	1.15	0.19	0.36	0.20	0.07	0.23
Basophil	0.0	0.00	0.00	0.00	0.00	0.00	0.00	0.00	0.00	0.00	0.00	0.00	0.00
Glucose	131.0	127.00	116.00	126.00	101.00	119.00	108.00	100.00	92.00	109.00	97.00	101.00	95.00
Urea Nitrogen	11.0	12.00	20.00	7.00	8.00	7.00	10.00	15.00	12.00	9.00	22.00	11.00	11.00
Creatinine	0.3	0.40	0.40	0.30	0.40	0.50	0.40	0.60	0.50	0.40	0.70	0.40	0.40
Sodium	141.0	144.00	143.00	144.00	141.00	143.00	144.00	145.00	145.00	145.00	143.00	145.00	144.00
Potassium	4.7	5.10	4.90	4.70	5.30	5.50	5.50	5.50	5.30	4.70	5.40	5.00	5.20
Chloride	102.0	102.00	101.00	105.00	101.00	103.00	106.00	106.00	108.00	108.00	106.00	108.00	108.00
Biocarbonate	22.0	25.00	21.00	21.00	20.00	21.00	19.00	19.00	17.00	21.00	19.00	20.00	18.00
Anion Gap	22.0	22.00	26.00	23.00	25.00	25.00	25.00	26.00	25.00	21.00	23.00	22.00	23.00
Albumin	2.9	3.00	3.10	2.90	2.80	3.00	2.60	2.50	2.70	2.80	2.80	2.60	2.70
Total Protein	5.5	5.50	5.40	5.30	5.30	5.50	4.90	4.80	5.00	5.30	5.20	5.00	5.10
Globulin	2.6	2.50	2.30	2.40	2.50	2.50	2.30	2.30	2.30	2.50	2.40	2.40	2.40
Calcium	***12.2***	11.30	10.80	11.20	10.40	11.60	11.00	10.80	11.10	10.80	10.60	10.70	10.70
Phosphorus	8.1	8.20	8.50	8.90	8.30	9.90	9.90	9.30	9.50	9.10	8.90	8.00	8.60
Magnesium	1.9	***2.10***	1.90	1.80	1.80	2.00	1.70	1.70	1.80	1.70	1.60	1.70	1.70
Cholesterol	370.0	296.00	243.00	224.00	263.00	282.00	232.00	234.00	232.00	261.00	257.00	291.00	238.00
Total Bilirubin	0.1	0.10	0.10	0.10	0.10	0.10	0.10	0.10	0.20	0.20	0.10	0.10	0.10
ALT	18.0	32.00	60.00	152.00	73.00	65.00	73.00	105.00	120.00	***130.00***	***133.00***	86.00	87.00
ALP	184.0	***279.00***	***339.00***	188.00	228.00	***239.00***	209.00	176.00	161.00	188.00	166.00	125.00	136.00
GGT	3.0	3.00	<3	<3	<3	3.00	<3	<3	***21.00***	3.00	3.00	3.00	<3
CK	378.0	312	530	500	526	593	1400	2590	***7667***	3835	***8798***	736	723

Basline: 1 week before injection

#, With immune suppression and AAV injection

†, With AAV injection

Black-bold-italic font, value above the VDML reference intervals.

Supplementary Fig. 1

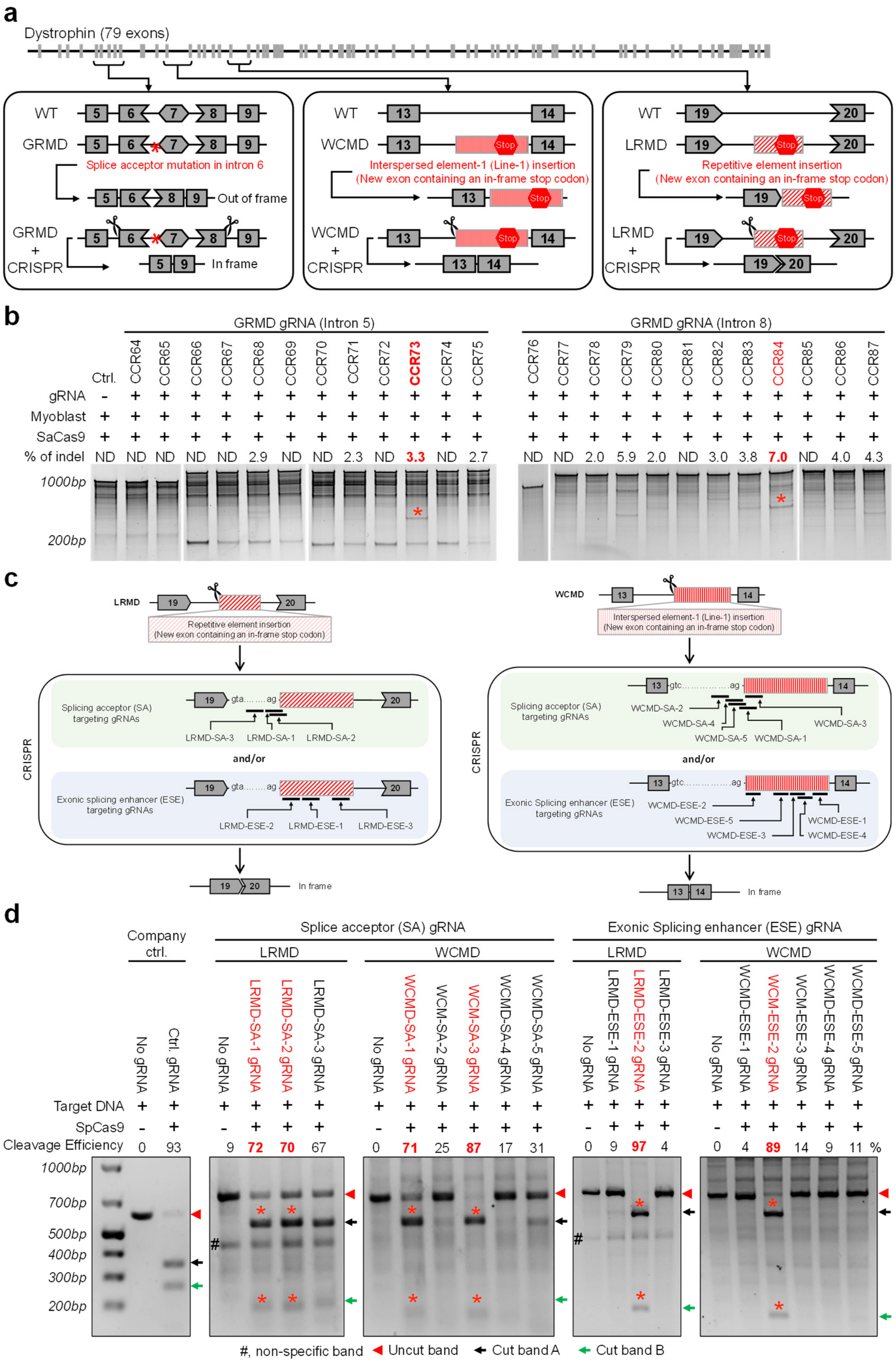

Supplementary Fig. 1. CRISPR editing strategies and *in vitro* screening. a, Cartoon illustration of the full-length dystrophin gene, mutations in golden retriever muscular dystrophy (GRMD), Welsh corgi muscular dystrophy (WCMD), and Labrador retriever muscular dystrophy (LRMD) models, and CRISPR strategies used to restore the reading frame in each model. **b,** Screening intron 5 and intron 8 gRNAs in primary myoblasts for editing the GRMD mutation. Left panel, intron 5 gRNA screening. *, the gRNA CCR73 displayed the highest activity with 3.3% indels. Right panel, intron 8 gRNA screening. *, the gRNA CCR84 displayed the highest activity with 7.0% indels. **c,** Cartoon illustration of the location of the candidate gRNAs designed for editing LRMD and WCMD mutations. **d,** Screening of gRNAs that target the splice acceptor and exonic splicing enhancer in LRMD and WCMD dogs. The target DNA for LRMD and WCMD was PCR-amplified using genomic DNA extracted from LRMD and WCMD muscles, respectively. The target control DNA was provided by the manufacturer. *, cut bands for gRNA with the highest cleavage efficiency.

Supplementary Fig. 2a,b

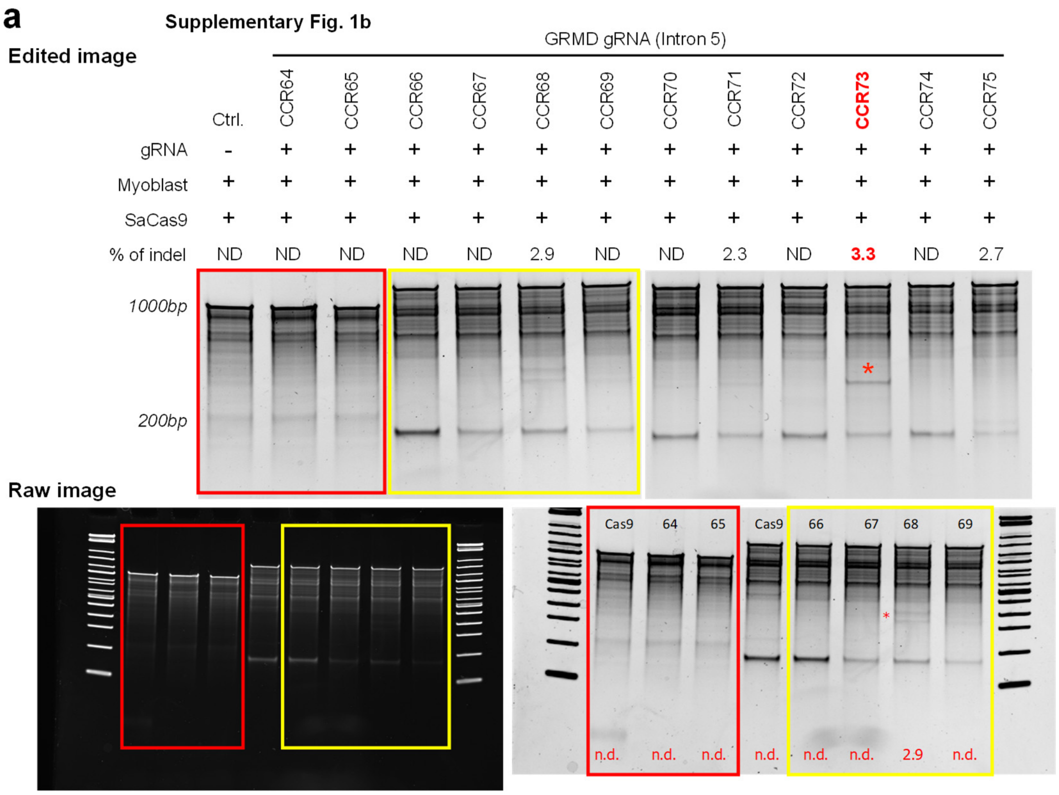

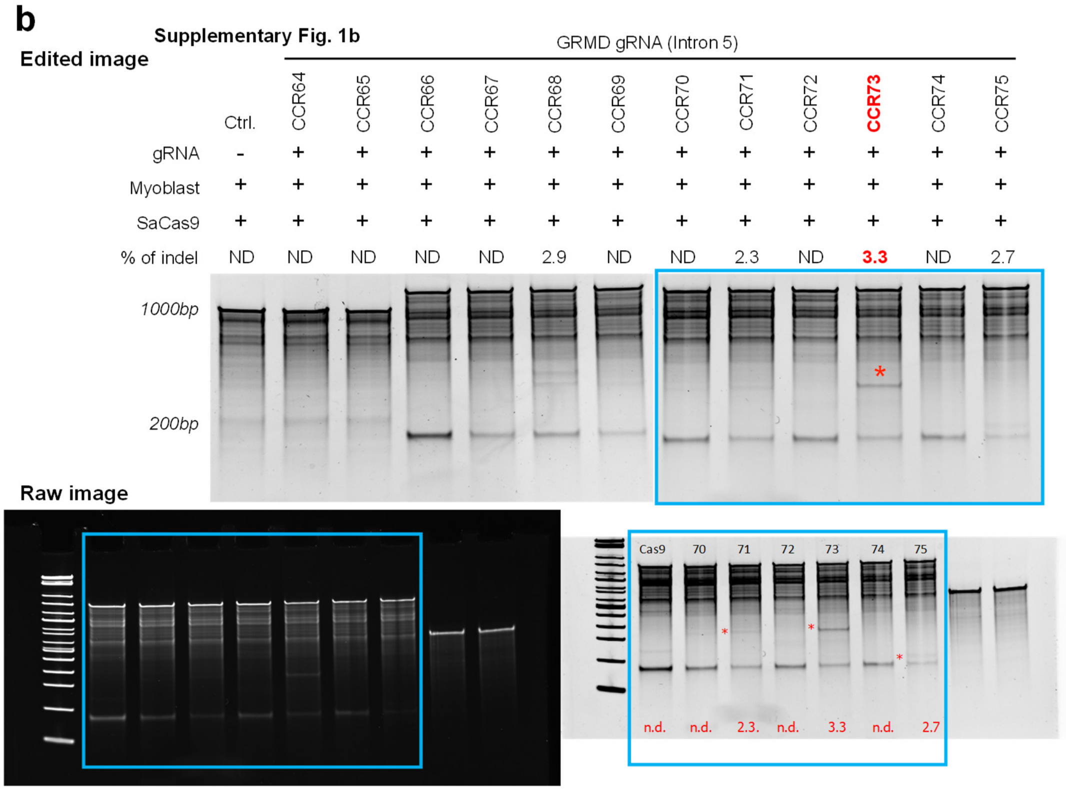

Supplementary Fig. 2c,d

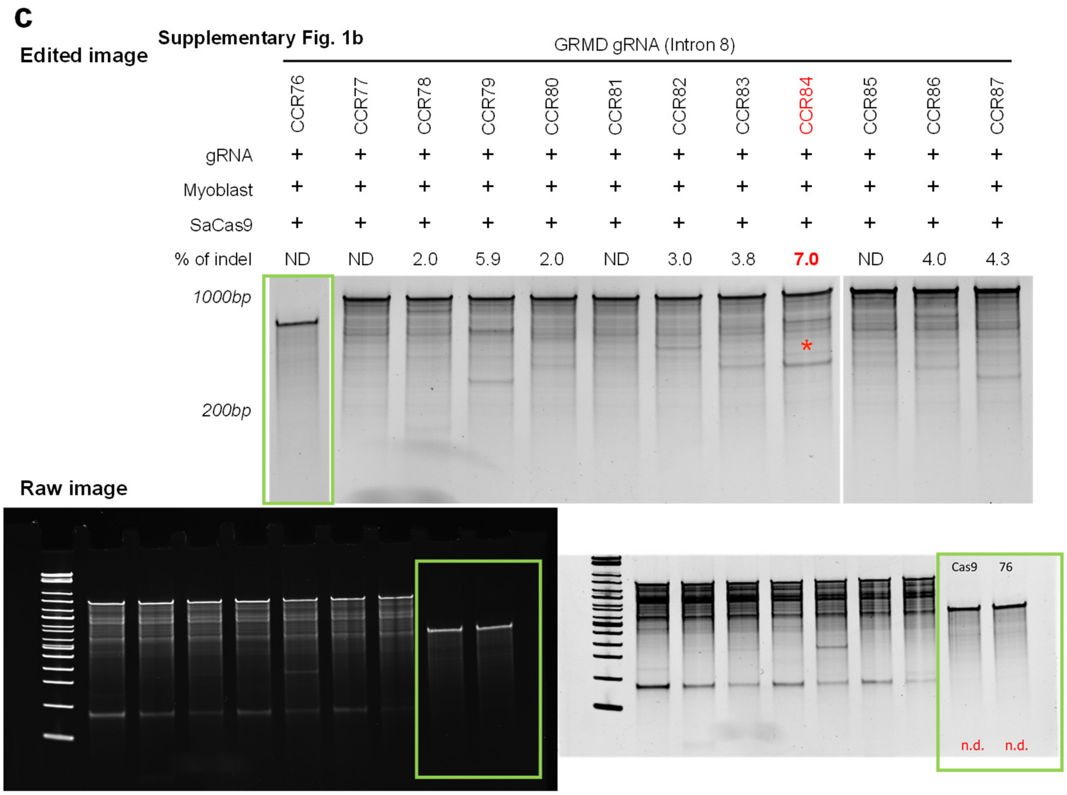

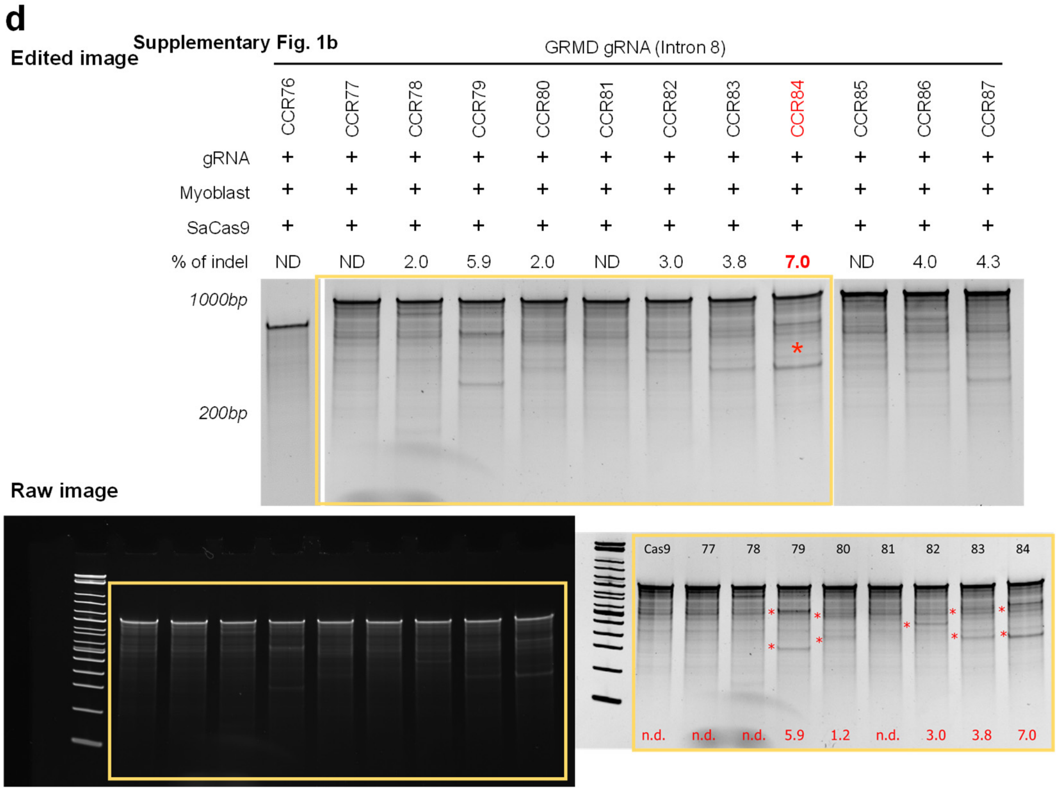

Supplementary Fig. 2e

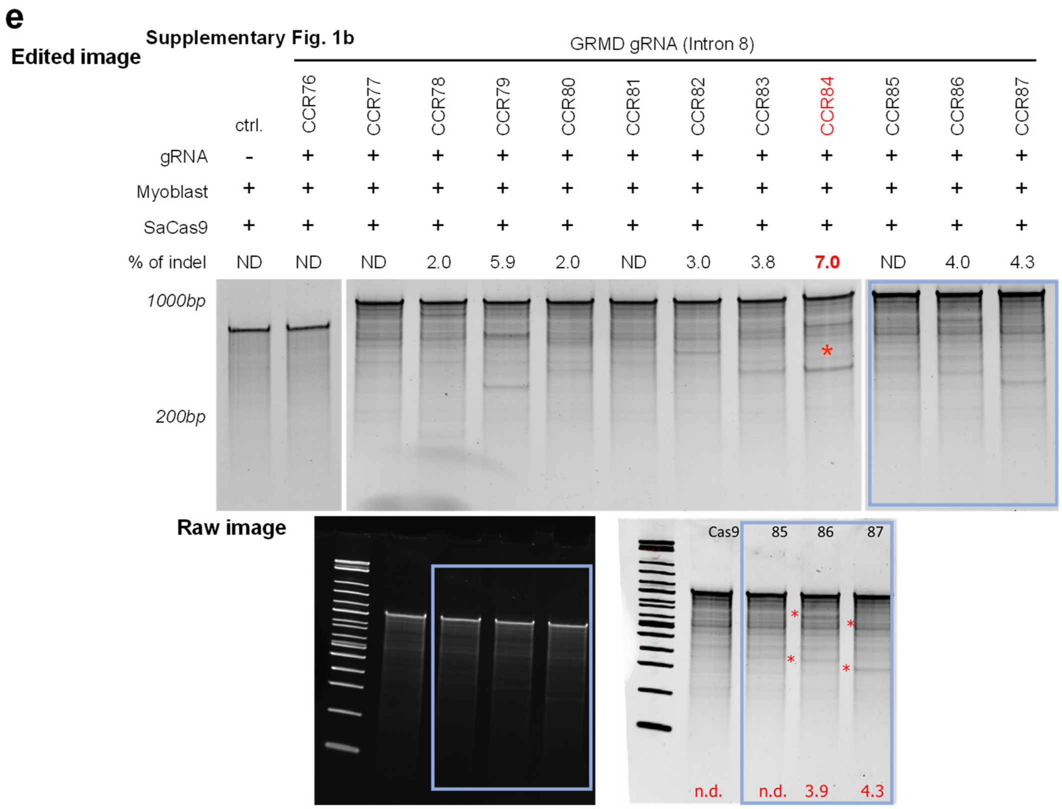

Supplementary Fig 2. Full-size unprocessed raw gel images and edited images for in vitro screening of gRNAs used in GRMD editing. **a,** Full-size raw and edited image for **Supplementary Fig. 1b** left panel CCR64 to CCR69 screening. **b,** Full-size raw and edited image for **Supplementary Fig. 1b** left panel CCR70 to CCR75 screening. **c,** Full-size raw and edited image for **Supplementary Fig. 1b** right panel CCR76 screening. **d,** Full-size raw and edited image for **Supplementary Fig. 1b** right panel CCR77 to CCR84 screening. **e,** Full-size raw and edited image for **Supplementary Fig. 1b** right panel CCR85 to CCR87 screening.

Supplementary Fig. 3a,b

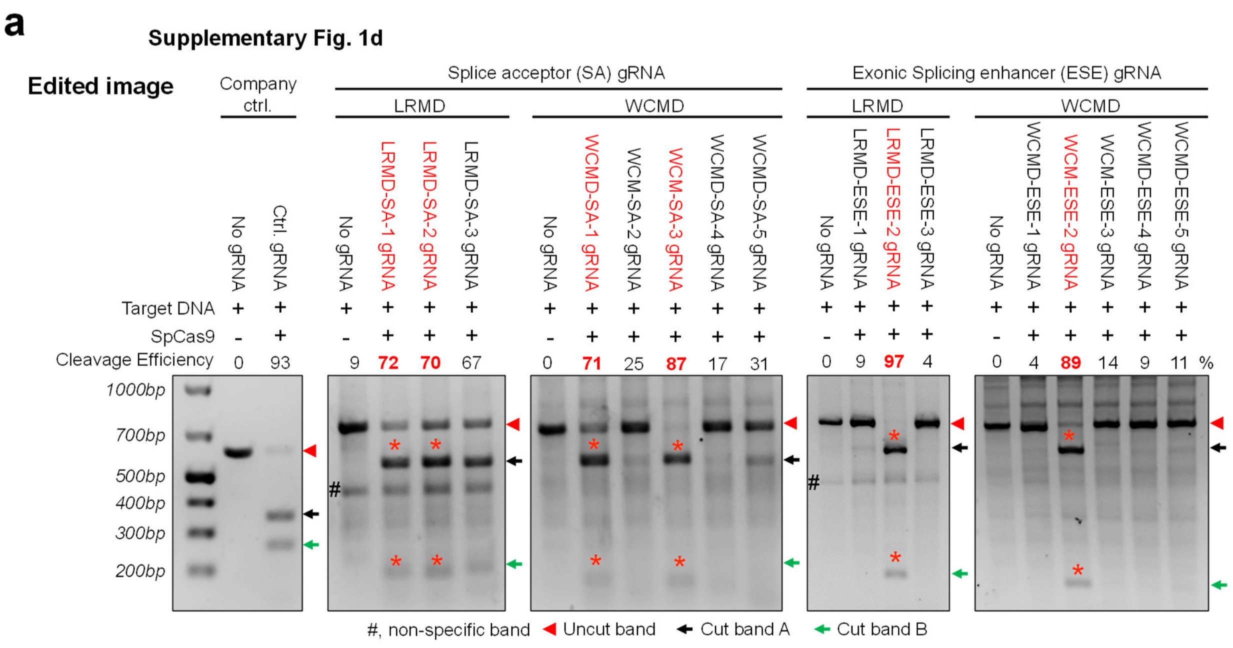

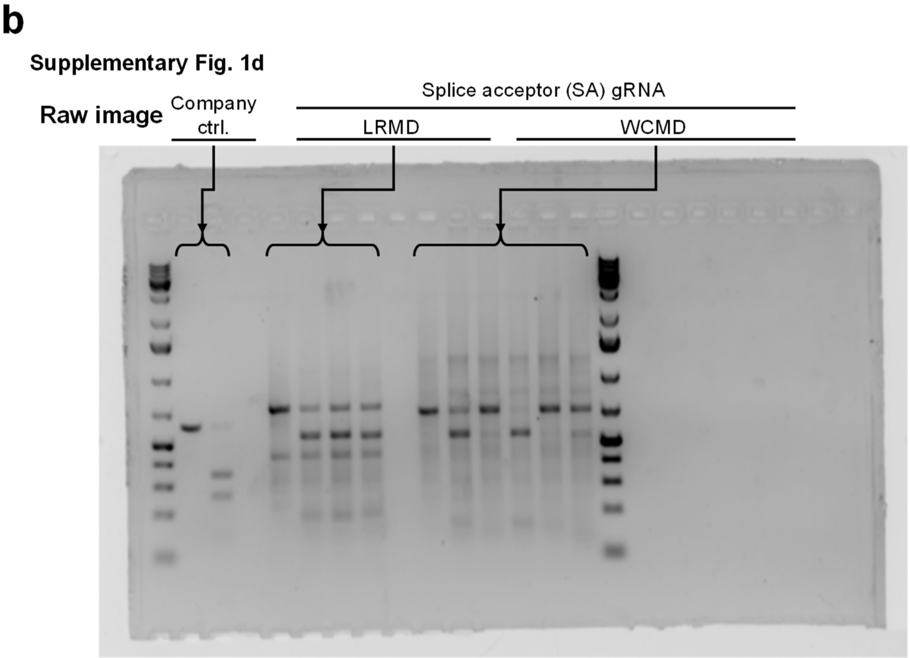

Supplementary Fig. 3c

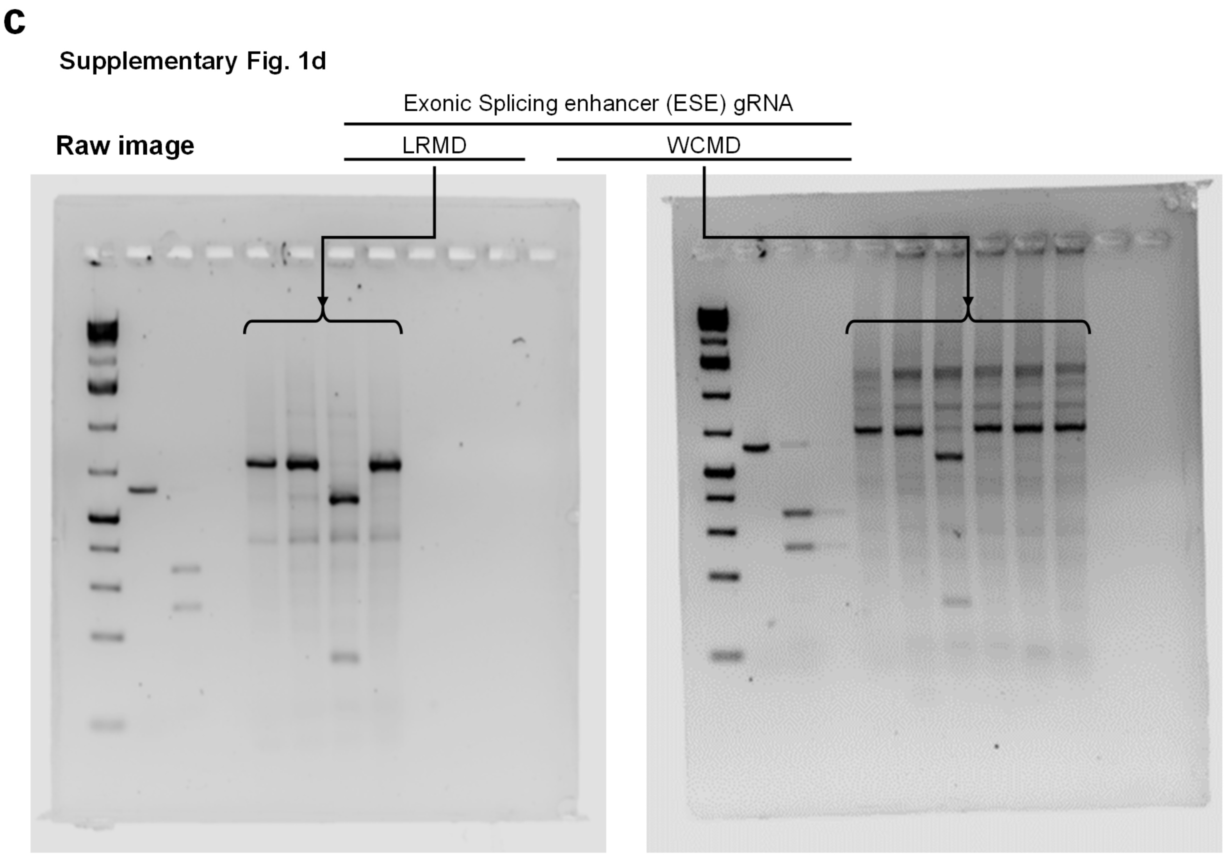

Supplementary Fig. 3. Full-size raw and edited images for in vitro screening of gRNAs used in WCMD and LRMD editing. a, Edited image shown in **Supplementary Fig. 1d**. **b,** Full-size raw image for splice acceptor gRNA screening. **c,** Full-size raw image for exonic splicing enhancer gRNA screening.

Supplementary Fig. 4

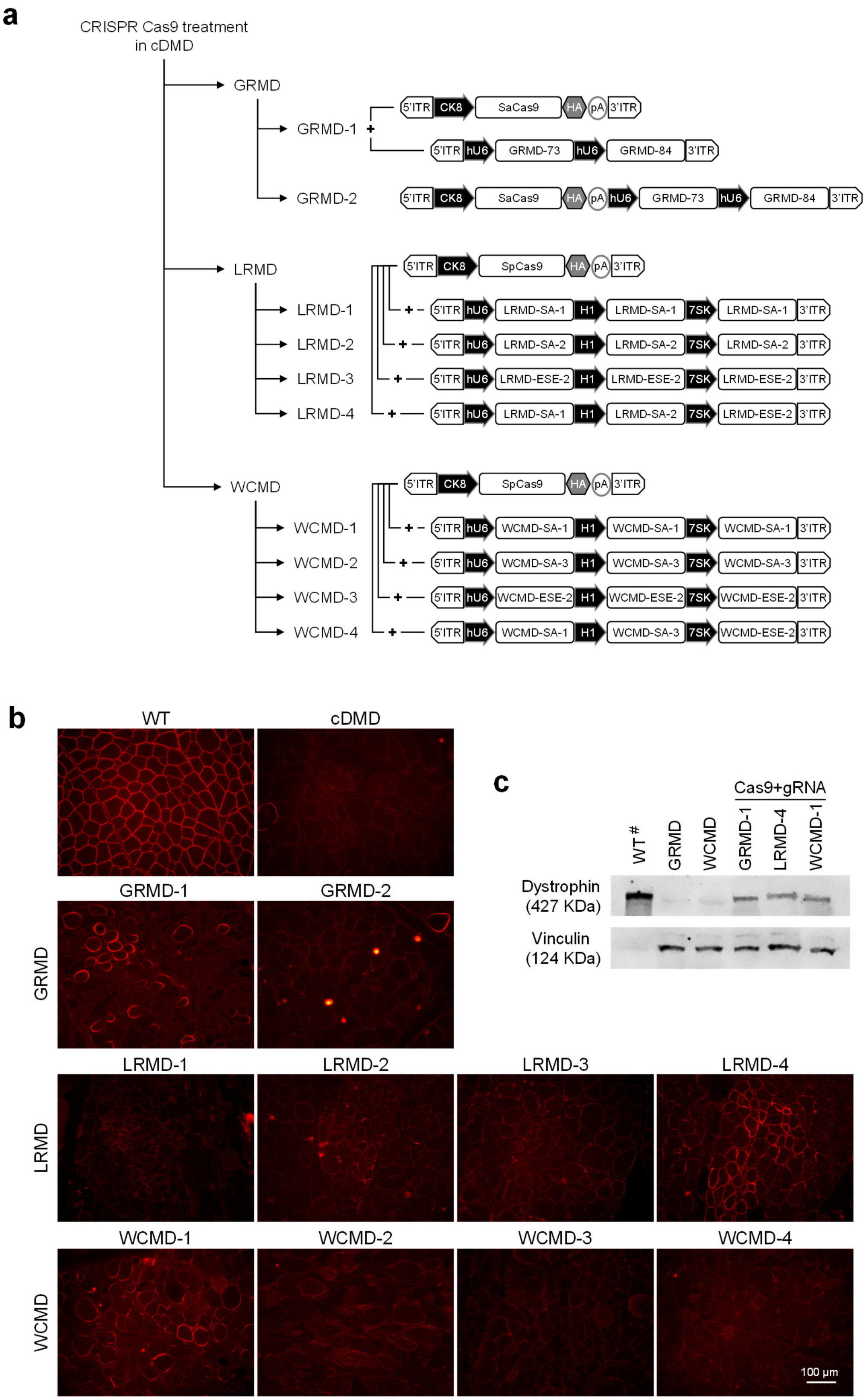

Supplementary Fig. 4. CRISPR editing AAV vectors and *in vivo* screening in affected dogs. **a,** Cartoon illustration of the AAV vectors for GRMD, LRMD, and WCMD editing. For GRMD editing, two approaches were used. GRMD-1 is a dual-vector approach in which SaCas9 and the gRNAs were expressed from two separate AAV vectors. GRMD-2 is a single-vector approach in which SaCas9 and the gRNAs were expressed from a single AAV vector. In both cases, SaCas9 was expressed from the muscle-specific CK8 promoter, while the gRNAs were expressed from the U6 promoter. The gRNAs used for GRMD editing are GRMD-73 and GRMD-84 (referred to as CCR73 and CCR84, respectively, in **Supplementary Table 1** and **Supplementary Fig. 1b**). For LRMD and WCMD editing, SpCas9 and the gRNAs were expressed from two separate AAV vectors. SpCas9 was expressed from the CK8 promoter, while the gRNAs were expressed from the human U6 (hU6), H1, and 7SK promoters. The gRNAs are marked in each vector **(Supplementary Fig. 1d**). SA, gRNA targeting the splice acceptor. ESE, gRNA targeting the exonic splicing enhancer. HA, HA-tag. pA, Polyadenylation. ITR, Inverted Terminal Repeat. **b and c,** Screening CRISPR editing AAV vectors in affected dogs by local injection. **b,** Representative dystrophin immunostaining from the muscle of a wild type (WT) dog, a non-injected affected (cDMD) dog, and AAV CRISPR treated GRMD, LRMD, and WCMD dogs at 3 weeks post-injection. **c,** Representative dystrophin western blot from WT, untreated affected dogs (GRMD and WCMD), and affected dogs treated with indicated CRISPR vectors. Vinculin was the loading control. #, WT loading is one-fourth of other dogs.

Supplementary Fig. 5a-c

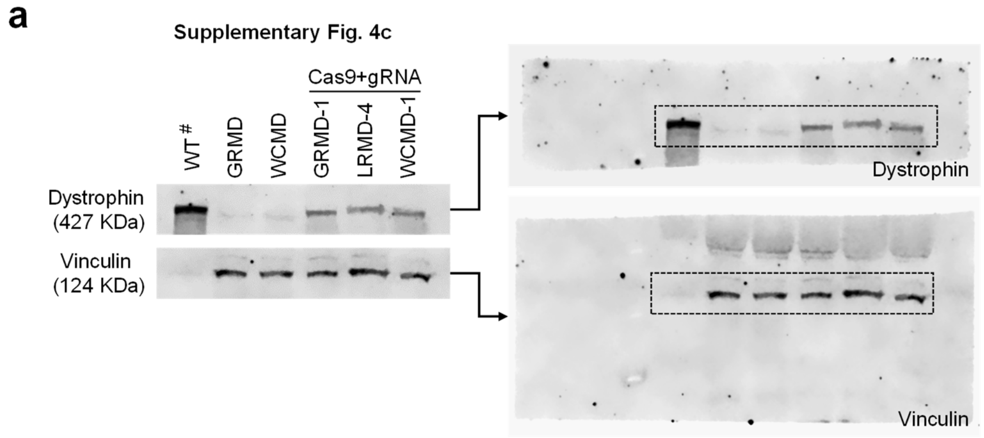

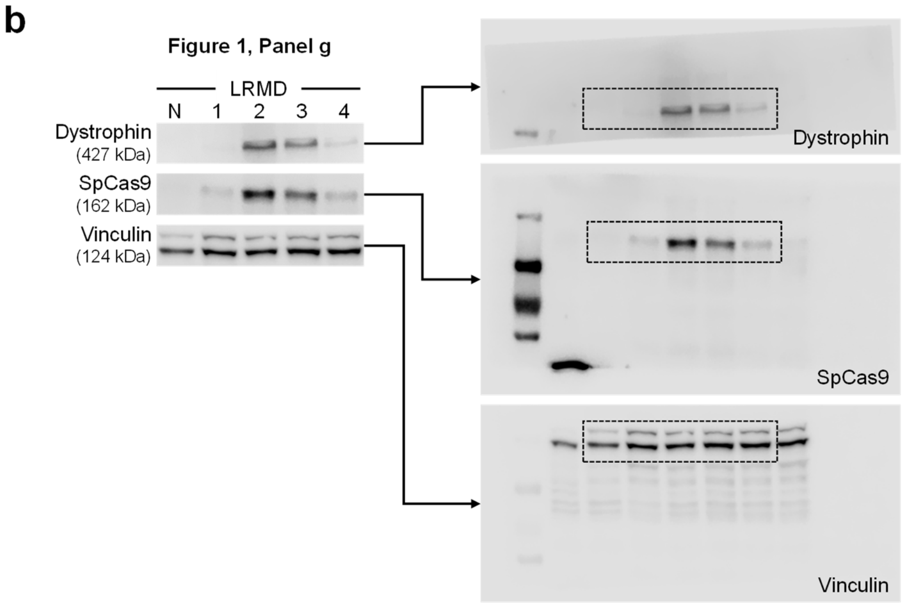

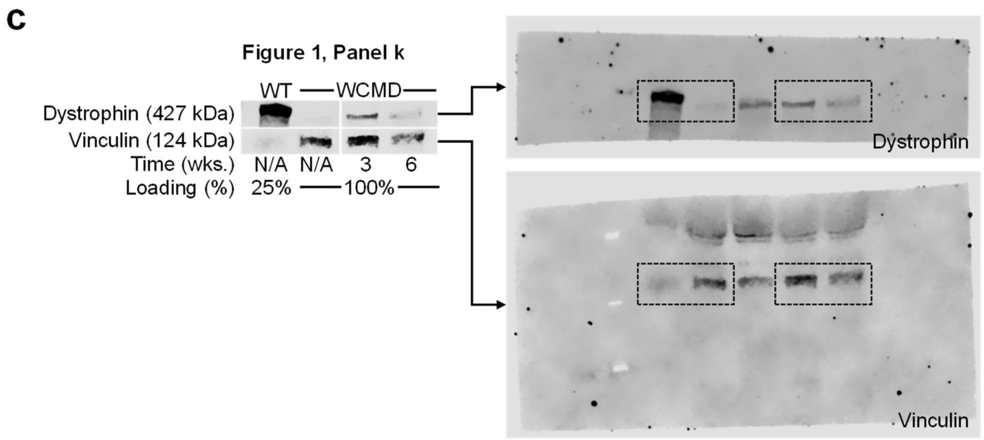

Supplementary Fig. 5d

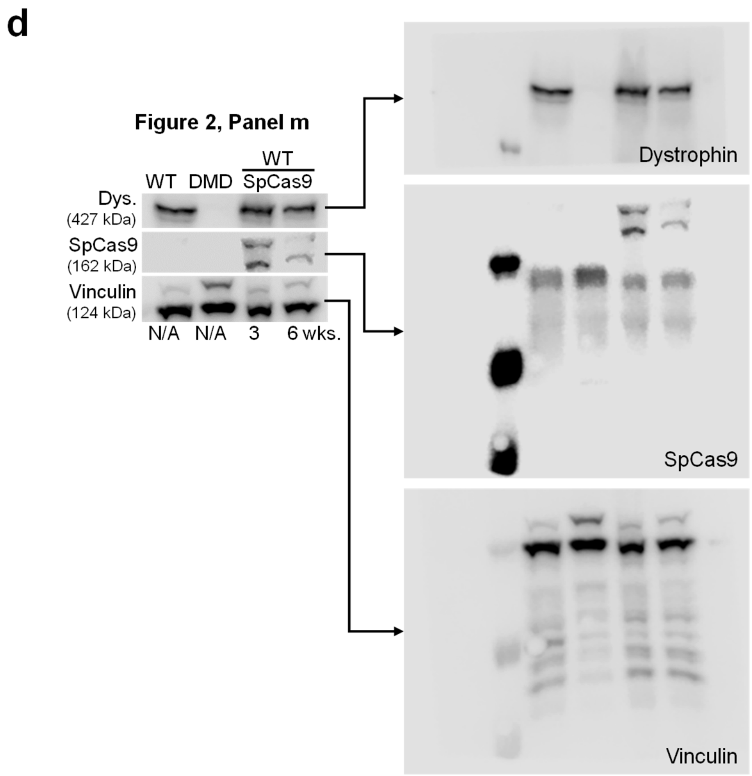

Supplementary Fig. 5. Raw and cropped western blot images. a, Western blot in Supplementary Figure 4 panel c. **b,** Western blot in Figure 1 panel g. **c,** Western blot in Figure 1 panel k. **d,** Western blot in Figure 2 panel m.

Supplementary Fig. 6

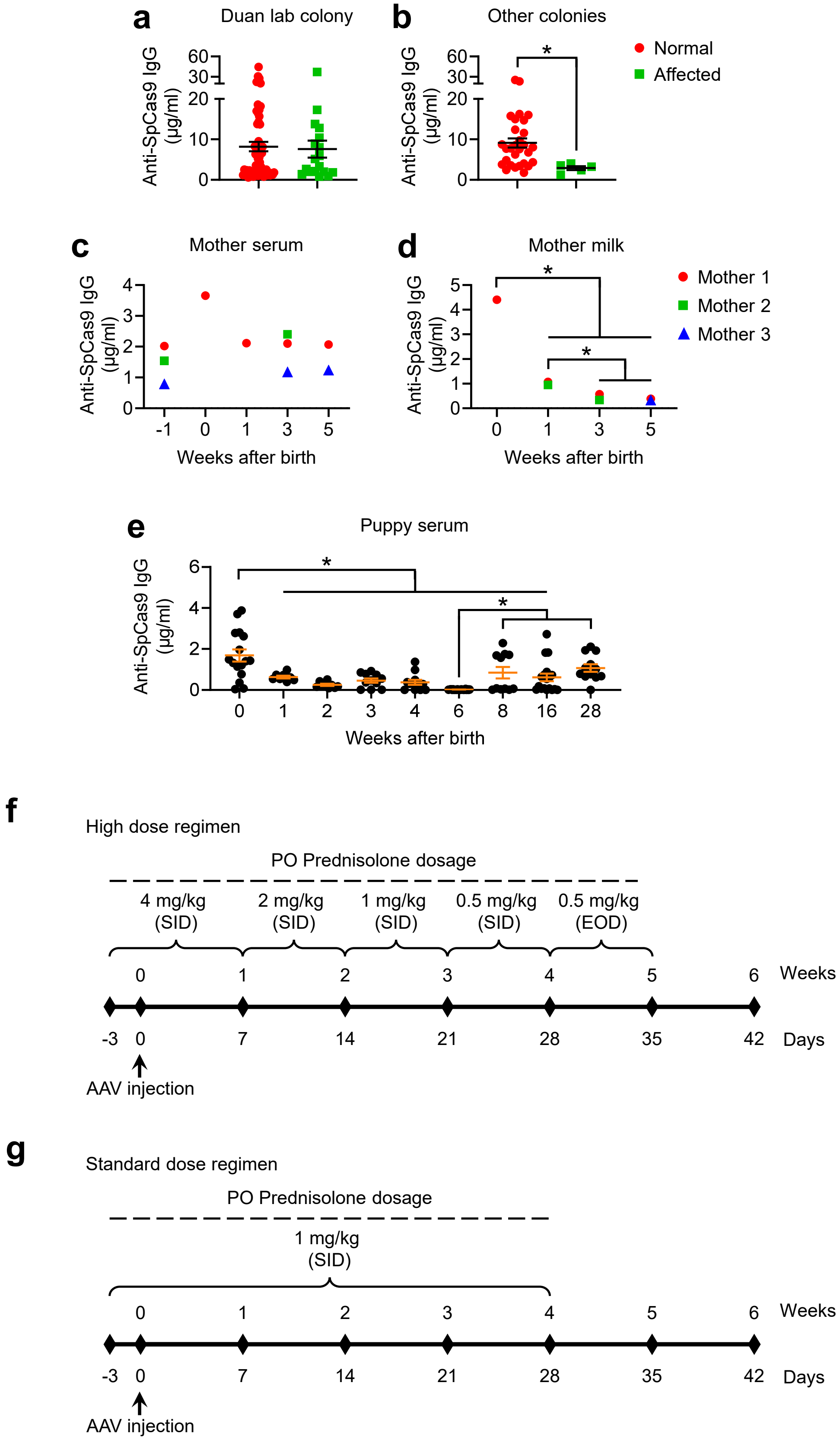

Supplementary Fig. 6. Cas9 antibody levels in naive dogs and immune suppression regimes used in the study. a, Serum anti-SpCas9 antibody levels in 9 to 48-m-old normal and affected dogs at the Duan laboratory colony (n=60 for normal dogs, n=18 for affected dogs). **b,** Serum anti-SpCas9 antibody levels in 4 to 80-m-old normal and affected dogs from other colonies (n=29 for normal dogs, n=5 for affected dogs). **c to e,** Serum anti-SpCas9 antibody from three litters. **c,** Antibody in mother's serum. **d,** Antibody in the milk. **e,** Antibody in puppy's serum (n=16, 8, 9, 11, 12, 23, 11, 18 and 12 for 0, 1, 2, 3, 4, 6, 8, 16 and 28 weeks after birth, respectively). Note, only a subset of puppies was bled between week 1 and week 28. **f,** High-dose prednisolone immune suppression regime. **g,** Standard-dose prednisolone immune suppression regime. PO, orally (per os); SID, Once a day; EOD, Every other day. Data are mean ± SEM. Statistical analysis was performed using One-way ANOVA with Tukey's multiple comparisons for **d-e,** and Student's t-test for **a-b**. See source data file for exact *p*-value. *, $p<0.05$.

Supplementary Fig. 7

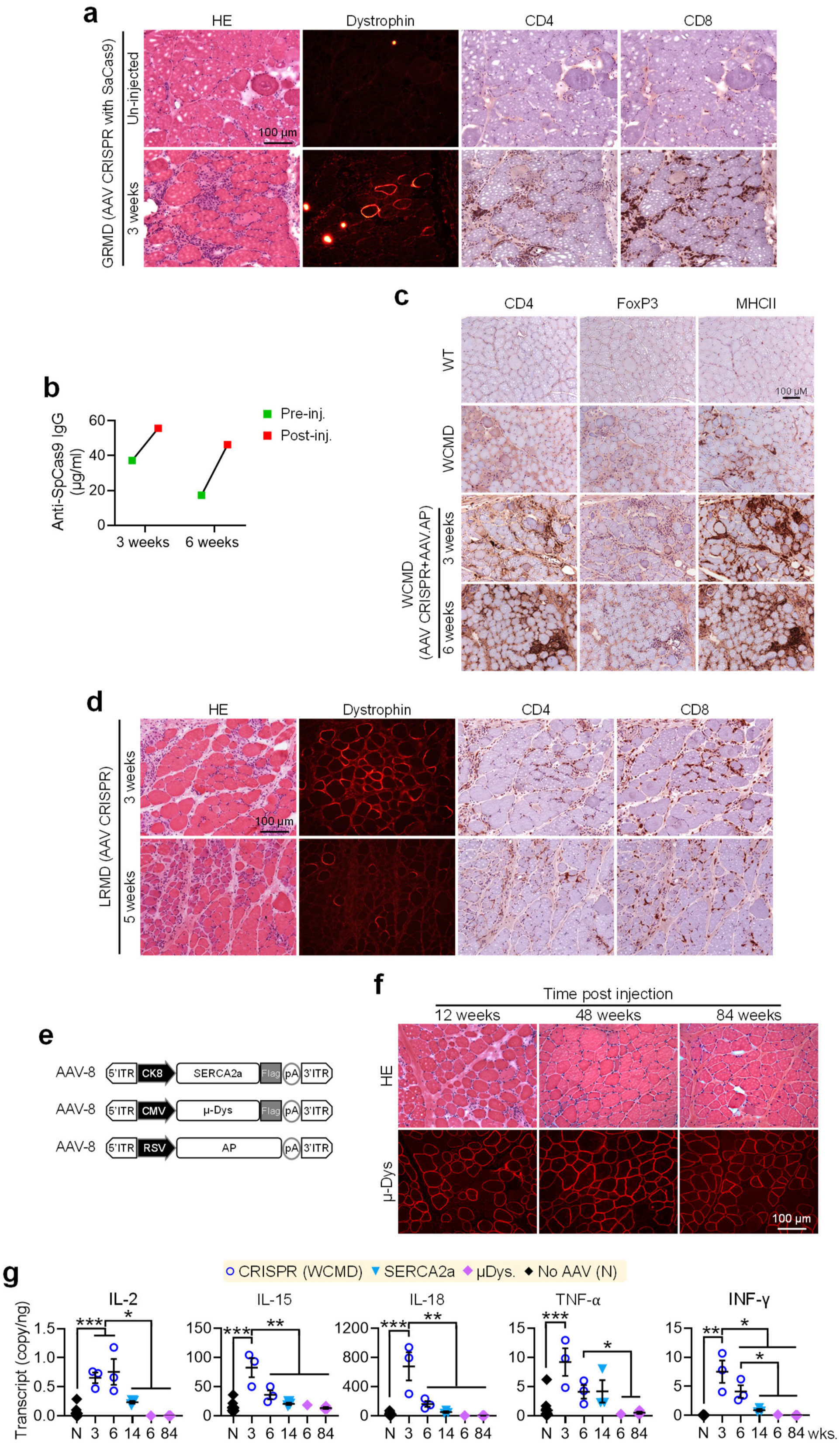

Supplementary Fig. 7. Additional data for local AAV injection in adult affected dogs. a, Representative HE staining, dystrophin, CD4, and CD8 immunostaining from an adult GRMD at 3 weeks after CRISPR therapy. An un-injected GRMD muscle was included as a control. **b,** Serum Cas9 antibody levels were increased in two 44-month-old WCMD dogs following AAV CRISPR therapy (n=1 per category). **c,** Representative CD4, FoxP3, and MHC II immunostaining from adult normal (WT), affected (WCMD), and CRISPR-treated WCMD dogs. FoxP3+ cells were not detected at 3 and 6 weeks post-CRISPR therapy. **d,** Representative 3-week and 5-week post-injection HE staining, dystrophin, CD4, and CD8 immunostaining from an adult LRMD dog that was treated with AAV CRISPR. **e,** Cartoon illustration of non-Cas9 vectors used in the study, including AAV.CK8.SERCA2a, AAV.CMV.micro-dystrophin, and AAV.RSV.AP. **f,** Representative HE staining and μ-dys immunostaining at 12-, 48- and 84-weeks after intramuscular injection of the AAV.CMV.μ-dys vector in an adult affected dog. **g,** Muscle cytokine levels from two adult WCMD dogs that were treated with intramuscular CRISPR editing, one adult affected that was treated with intramuscular SERCA2a therapy, and one adult affected that was treated with intramuscular micro-dystrophin therapy (N, n= 14; n=3 for each category except n=1 for μ-Dys 6 wks). N, No AAV. Data are mean ± SEM. Statistical analysis was performed using One-way ANOVA with Tukey's multiple comparisons for **g**. See source data file for the exact *p*-value. *, $p<0.05$; **, $p<0.01$; ***, $p<0.001$.

Supplementary Fig. 8

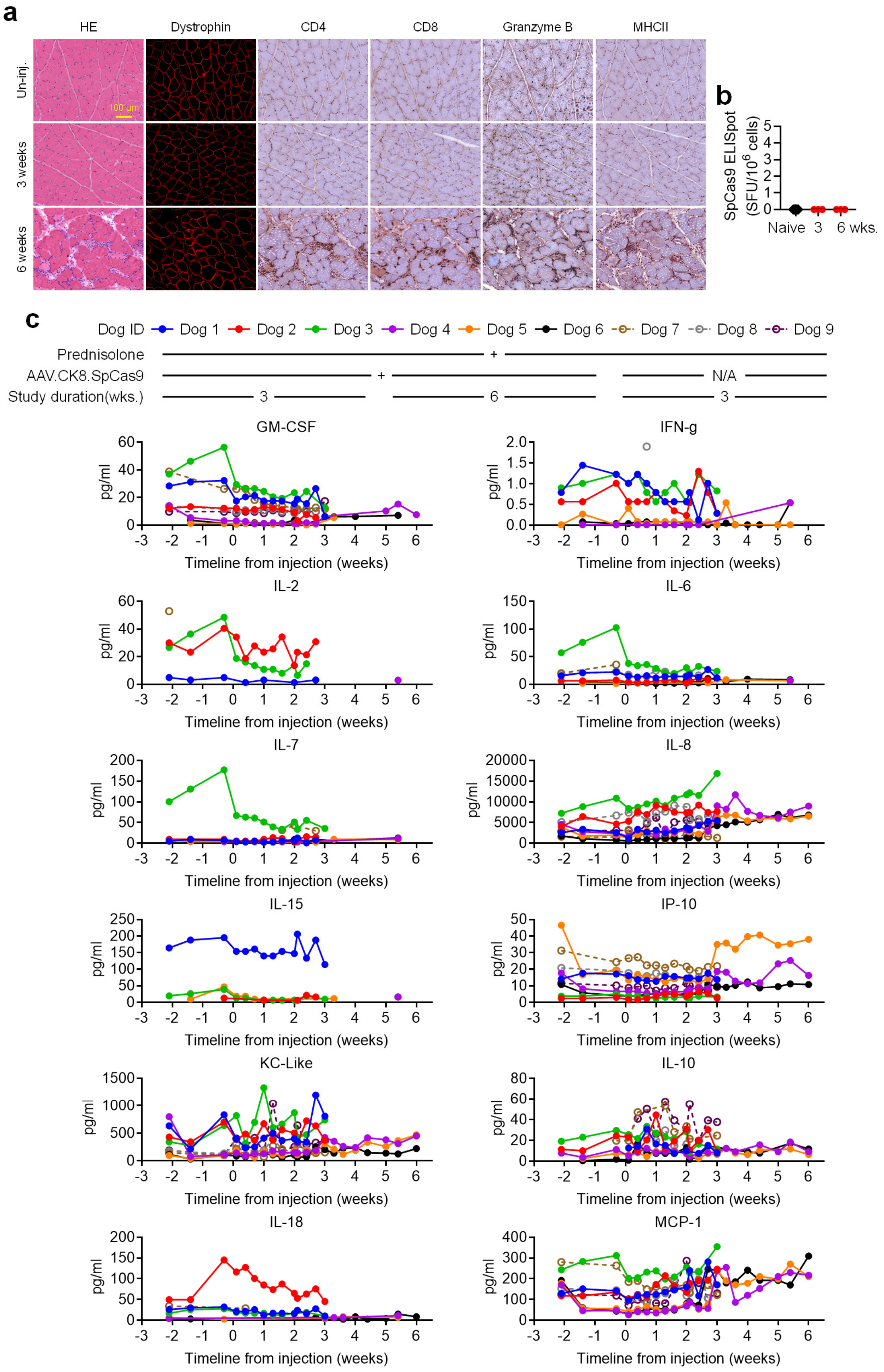

Supplementary Fig. 8. Additional data for local AAV.CK8.SpCas9 injection in adult normal dogs. a, Representative HE staining, dystrophin, CD4, CD8, granzyme B, and MHCII immunostaining at 3 and 6 weeks after injection. Un-inj., a muscle that did not receive AAV injection. **b**, Cas9-specific IFN- γ ELISpot assay on PBMCs (Naïve, n=6; others, n=3). **c,** Serum cytokine changes from normal adult dogs that received immune suppression only or both immune suppression and intramuscular AAV.CK8.SpCas9 injection. Each line represents data from one dog at the indicated time point.

Supplementary Fig. 9

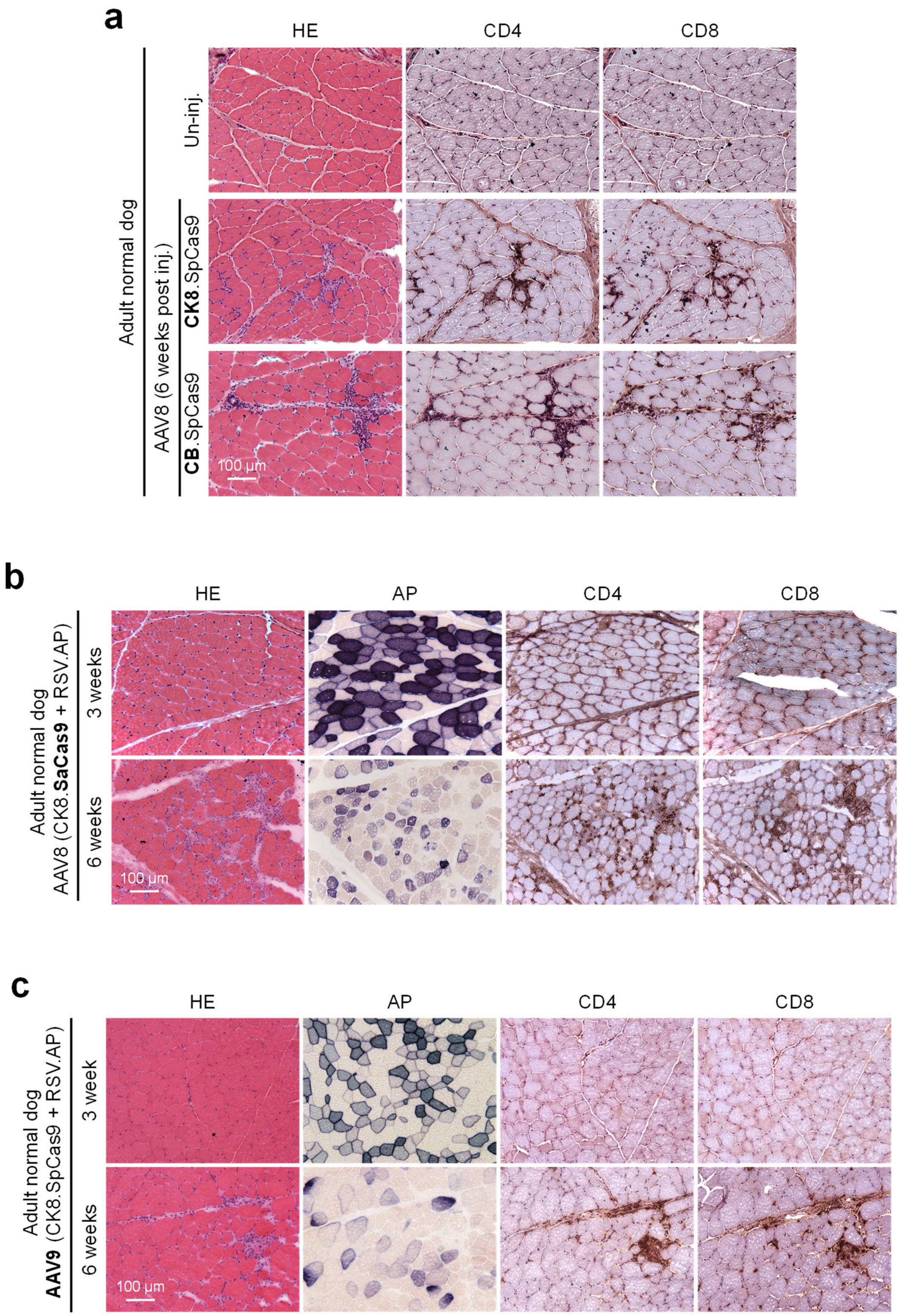

Supplementary Fig. 9. The Cas9-induced cellular immune response is independent of the promoter, bacterial species of Cas9, and AAV serotype. a, Representative 6-week muscle biopsy HE staining, CD4 and CD8 immunostaining from normal dogs that received AAV8 vectors carrying either the CK8.SpCas9 expression cassette or the CB.SpCas9 expression cassette. A non-injected (un-inj.) normal dog muscle was included as a control. CK8, muscle-specific creatine kinase 8 promoter; CB, chicken β-actin promoter. **b,** Representative 3-week and 6-week muscle biopsy HE and AP staining, CD4 and CD8 immunostaining from an adult normal dog that was co-injected with an AAV8 CK8.SaCas9 vector and an AAV8 RSV.AP vector. **c,** Representative 3-week, and 6-week muscle biopsy HE and AP staining, CD4 and CD8 immunostaining from an adult normal dog that was co-injected with an AAV9 CK8.SpCas9 vector and an AAV9 RSV.AP vector.

Supplementary Fig. 10

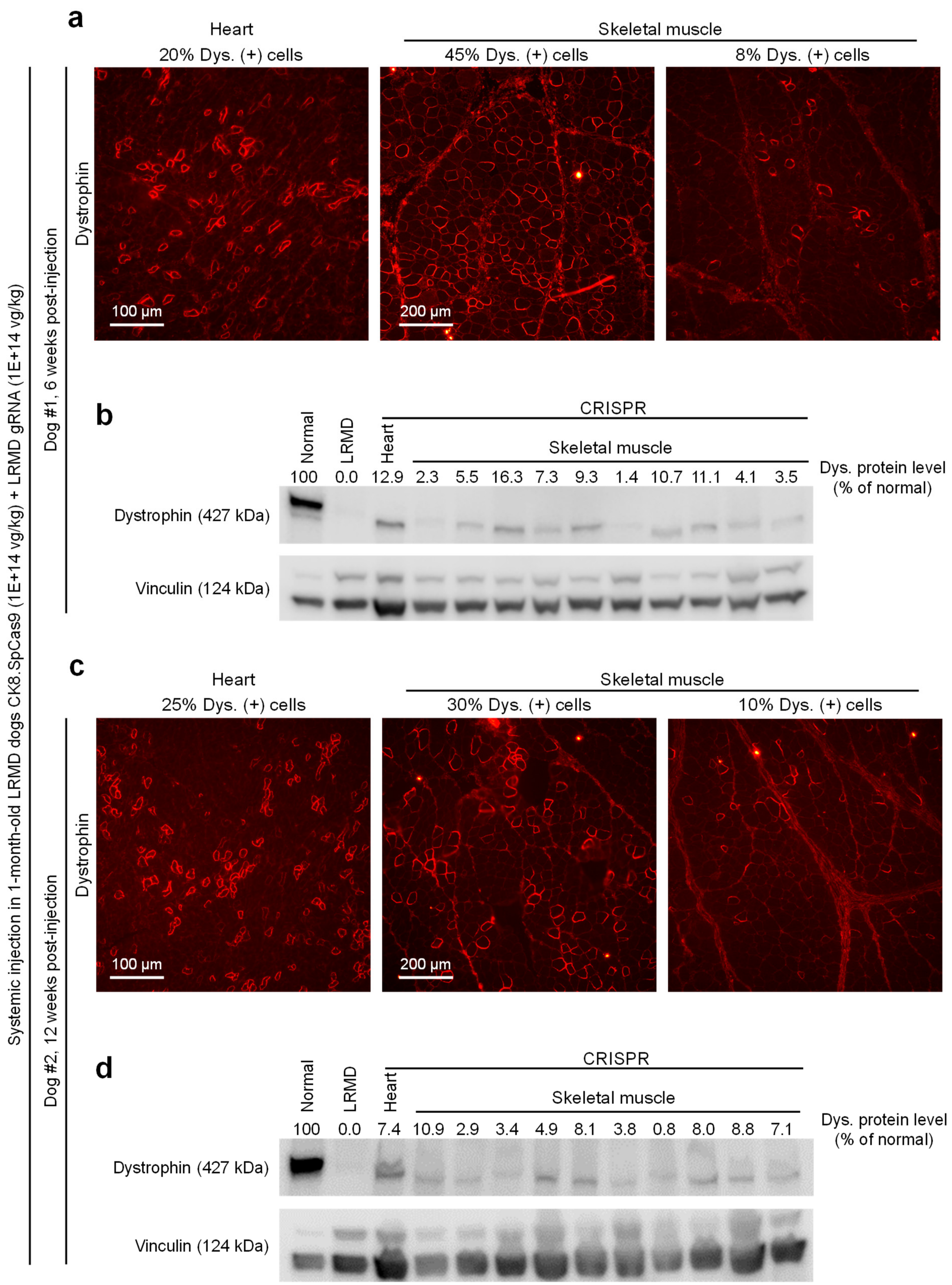

Supplementary Fig. 10. Representative dystrophin immunostaining and western blots from LRMD dogs that received systemic AAV CRISPR therapy at 1 month of age. a, Dystrophin immunostaining of the heart and two skeletal muscles from dog #1 (harvested at 6 weeks post-injection). The percent of dystrophin positive cells is marked in each image. **b,** Representative dystrophin western blot from dog #1. The level of dystrophin expression is marked for each lane. **c,** Dystrophin immunostaining of the heart and two skeletal muscles from dog #2 (harvested at 12 weeks post-injection). The percent of dystrophin positive cells is marked in each image. **d,** Representative dystrophin western blot from dog #2. The level of dystrophin expression is marked for each lane.

Supplementary Fig. 11

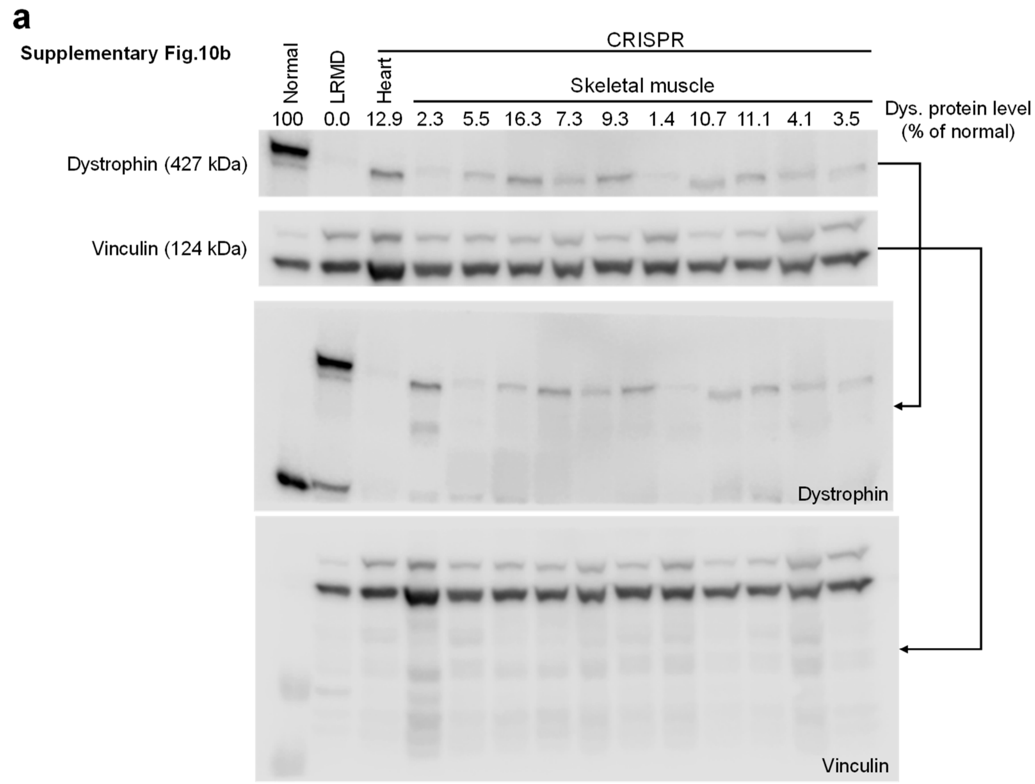

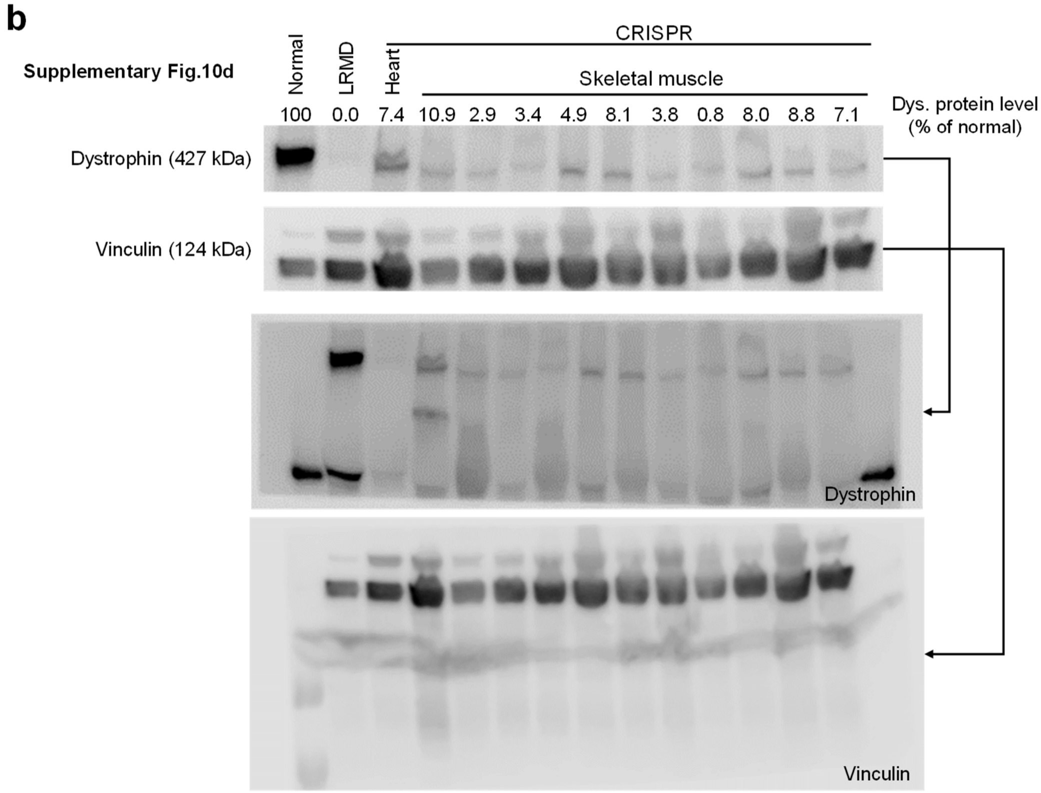

Supplementary Fig. 11. Dystrophin western blots from systemic AAV CRISPR treated dogs. a, Raw and cropped western blot images in Supplementary Fig. 10b. **b,** Raw and cropped western blot images in Supplementary Fig. 10d.

Supplementary Fig. 12

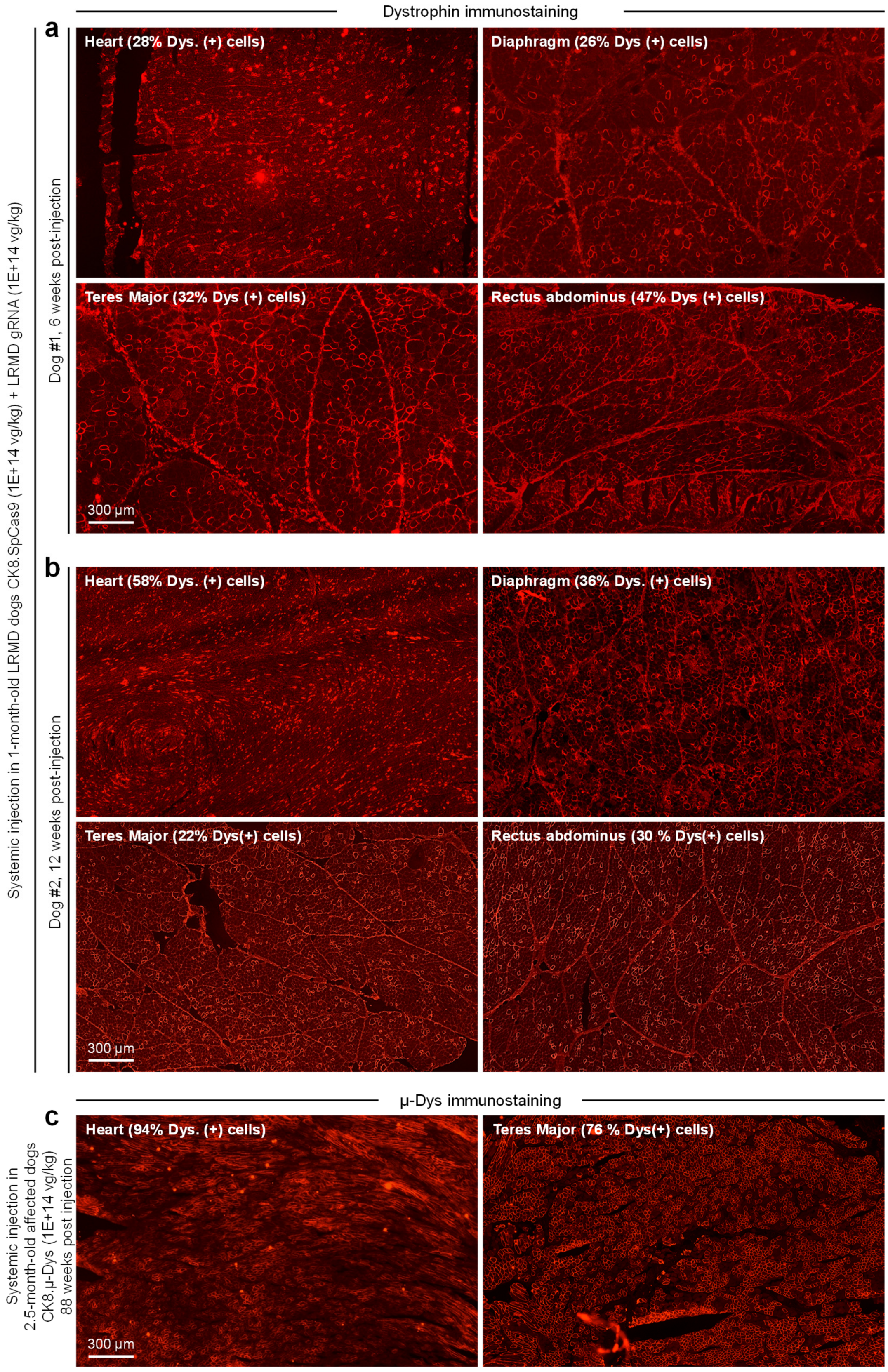

Supplementary Fig. 12. Representative full-view dystrophin immunostaining photomicrographs from systemic AAV CRISPR treated dogs and the systemic AAV micro-dystrophin treated dog. a, Heart, diaphragm, teres major, and rectus abdominus from CRISPR-treated dog #1 (injected at 1 month of age and harvested at 6 weeks post-injection). **b,** Heart, diaphragm, teres major, and rectus abdominus from CRISPR-treated dog #2 (injected at 1 month of age and harvested at 12 weeks post-injection). **c,** Heart and teres major from an affected dog that received systemic AAV.micro-dystrophin (CK8.μ-Dys) therapy at 2.5 months of age and harvested at 88 weeks post-injection. The percent of dystrophin positive cells is marked in each image.

Supplementary Fig. 13

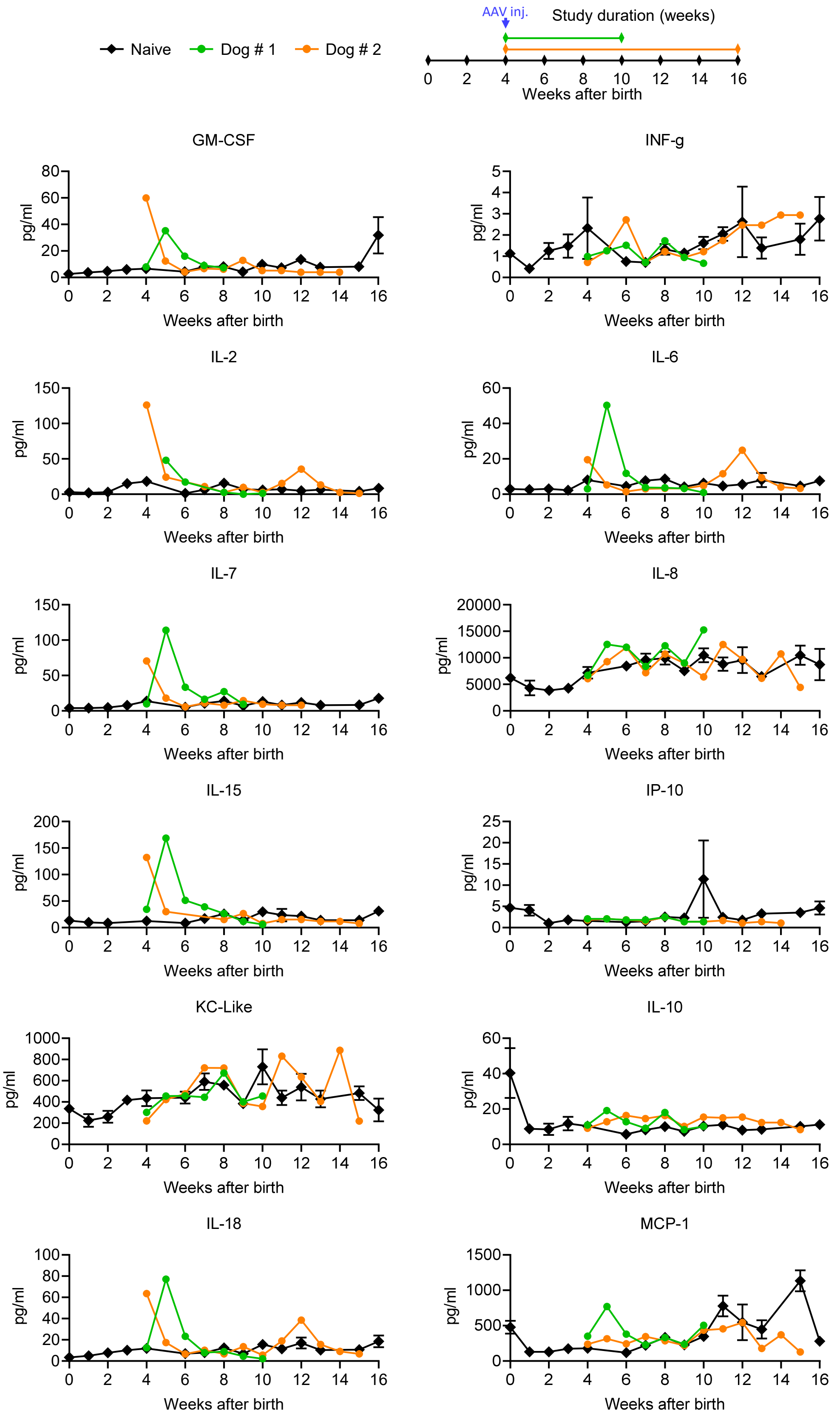

Supplementary Fig. 13. Serum cytokines from LRMD dogs that received systemic AAV CRISPR therapy at 1 month of age. Black, data from untreated naïve dogs. Green, a LRMD dog that was treated at 4 weeks of age and euthanized at 10 weeks of age. Orange, LRMD dog that was treated at 4 weeks of age and euthanized at 16 weeks of age. Each panel depicts the data for one cytokine as indicated. Each colored line represents data from one dog at the indicated time point. The black line represents data from naïve dogs (n=20 to 3).

Supplementary Fig. 14

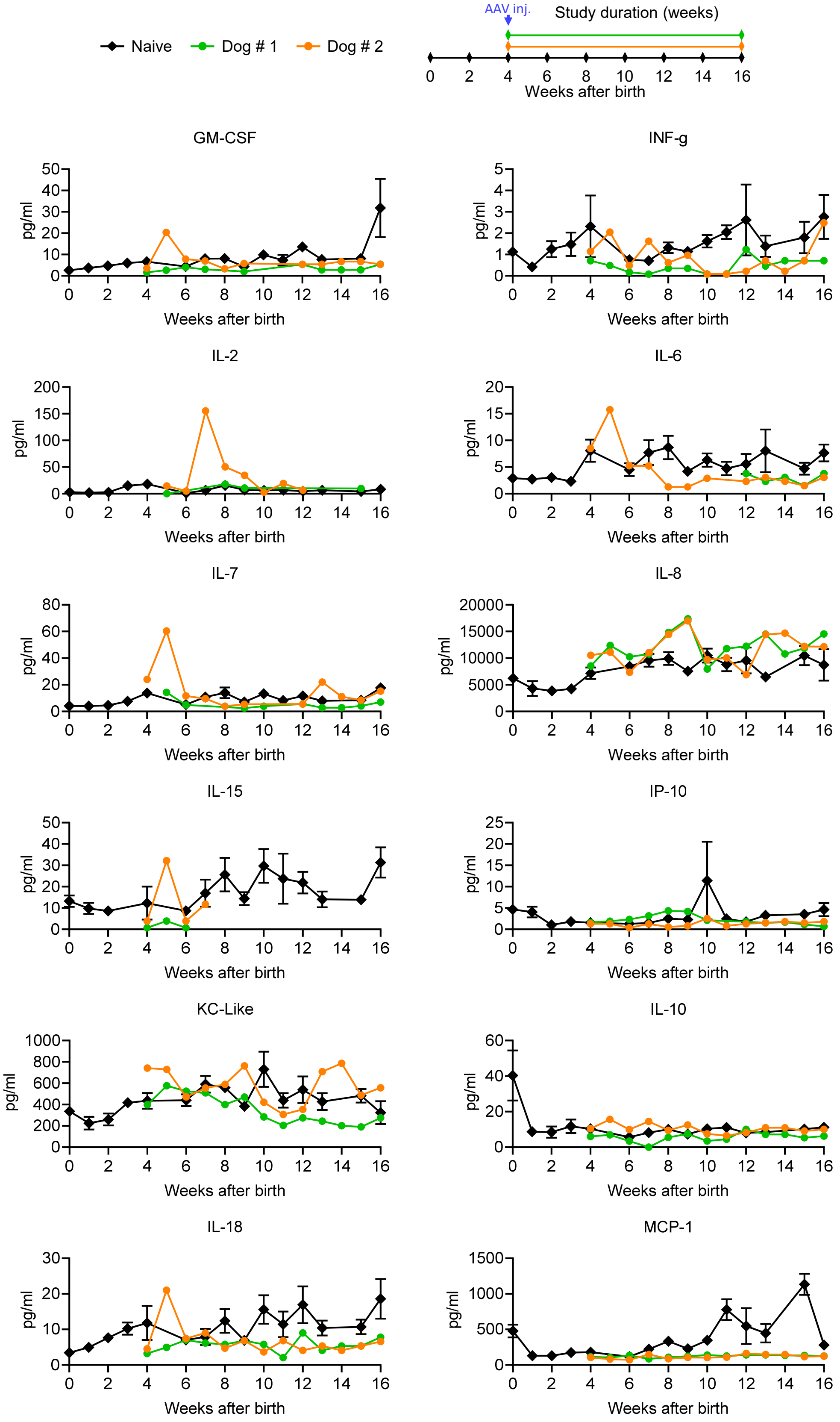

Supplementary Fig. 14. Serum cytokines from normal dogs that received systemic AAV.CK8.SpCas9 injection at 1 month of age. Black, data from untreated naïve dogs. Green, a normal dog that was treated at 4 weeks of age and euthanized at 16 weeks of age. Orange, another normal dog that was treated at 4 weeks of age and euthanized at 16 weeks of age. Each panel depicts the data for one cytokine as indicated. Each colored line represents data from one dog at the indicated time point. The black line represents data from naïve dogs (n=20 to 3).

Supplementary Fig. 15

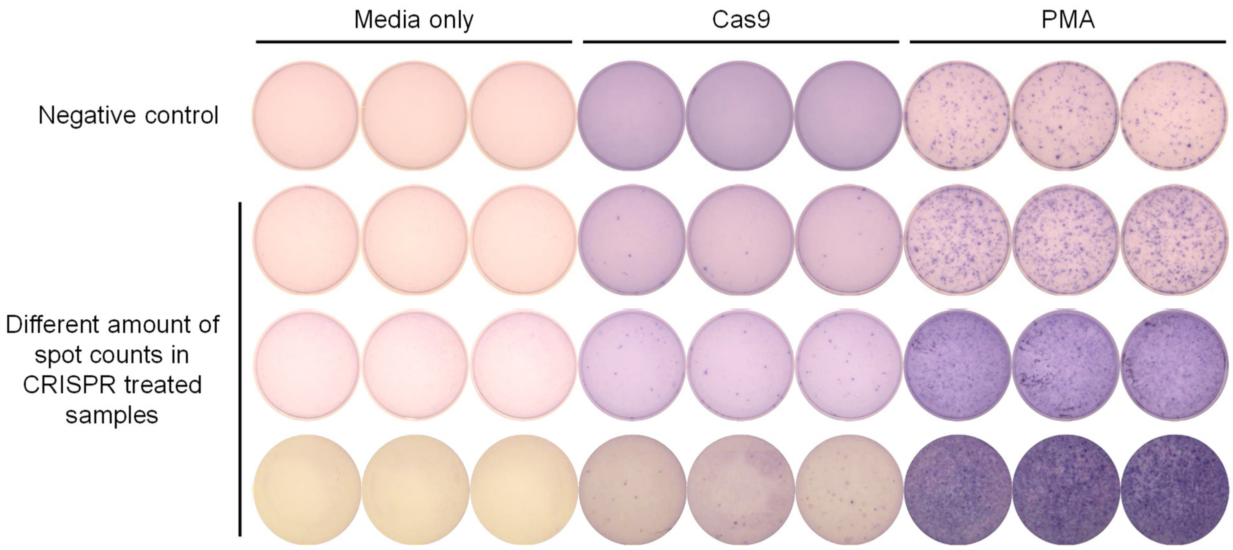

Supplementary Fig.15. Representative ELISpot images from an untreated dog (negative control), three CRISPR-treated dogs, and PMA controls (positive control). Media controls were included in each assay as additional negative controls. Assays were performed in triplicates. No spot was detected in negative controls.
