# Supplementary material for: Cas9-specific immune responses compromise local and systemic AAV CRISPR therapy in multiple dystrophic canine models
Source: Nat Commun. 2021 Nov 24;12:6769. doi: 10.1038/s41467-021-26830-7 (PMC8613397; doi:10.1038/s41467-021-26830-7)
Supplement: Supplementary file 3 — Reporting Summary [file 41467_2021_26830_MOESM3_ESM.pdf]

nature portfolio

Corresponding author(s): Dongsheng Duan

Last updated by author(s): 08-13-21

Reporting Summary

Nature Portfolio wishes to improve the reproducibility of the work that we publish. This form provides structure for consistency and transparency in reporting. For further information on Nature Portfolio policies, see our Editorial Policies and the Editorial Policy Checklist.

Statistics

For all statistical analyses, confirm that the following items are present in the figure legend, table legend, main text, or Methods section.

n/a	Confirmed	
☐	☒	The exact sample size (*n*) for each experimental group/condition, given as a discrete number and unit of measurement
☐	☒	A statement on whether measurements were taken from distinct samples or whether the same sample was measured repeatedly
☐	☒	The statistical test(s) used AND whether they are one- or two-sided<br>*Only common tests should be described solely by name; describe more complex techniques in the Methods section.*
☒	☐	A description of all covariates tested
☐	☒	A description of any assumptions or corrections, such as tests of normality and adjustment for multiple comparisons
☐	☒	A full description of the statistical parameters including central tendency (e.g. means) or other basic estimates (e.g. regression coefficient) AND variation (e.g. standard deviation) or associated estimates of uncertainty (e.g. confidence intervals)
☐	☒	For null hypothesis testing, the test statistic (e.g. *F*, *t*, *r*) with confidence intervals, effect sizes, degrees of freedom and *P* value noted<br>*Give P values as exact values whenever suitable.*
☒	☐	For Bayesian analysis, information on the choice of priors and Markov chain Monte Carlo settings
☒	☐	For hierarchical and complex designs, identification of the appropriate level for tests and full reporting of outcomes
☒	☐	Estimates of effect sizes (e.g. Cohen's *d*, Pearson's *r*), indicating how they were calculated

Our web collection on statistics for biologists contains articles on many of the points above.

Software and code

Policy information about availability of computer code

Data collection

*Experimental dogs: No software was used.
*Design of the gRNA for GRMD editing: No software was used.
*Surveyor screening for GRMD editing gRNAs: Image Lab software (Version 3.0, Bio-Rad).
*Design of single gRNA for WCMD and LRMD mutations: No software was used.
*In vitro screening of the sgRNA for LRMD and WCMD editing: No software was used.
*The gRNA cis-plasmids for the GRMD editing: No software was used.
*The gRNA cis-plasmids for the LRMD and WCMD editing: No software was used.
*AAV production and purification: No software was used.
*Transient immune suppression: No software was used.
*AAV administration: No software was used.
*Morphological analysis: Leica Application Suite software (Version 4.12.0, Leica Camera Inc.).
*Western blot: Image Studio™ Lite Software (Version 5.2.5, LI-COR Inc.)
*Vector genome copy number quantification: Sequence Detection System (SDS) software (Version 2.4, ThermoFisher Scientific).
*Vector transcript quantification: Sequence Detection System (SDS) software (Version 2.4, ThermoFisher Scientific).
*Cytokine transcript quantification: QuantaSoft software (Version 1.0, Bio-Rad).
*Anti-SpCas9 antibody assay: Gen5 software (Version 3.04, BioTeck): ImmunoSpot (Version 7, Cellular Technology Limited)
*Serum Cytokine quantification: Luminex XPONENT software (Version 4.2, Luminex Corp.).
*Blood chemistry: No software was used.
*Statistical analysis: No software was used.

Data analysis

*Experimental dogs: No software was used.
*Design of the gRNA for GRMD editing: Cas-OFFfinder software (Version (no data), http://www.rgenome.net/cas-offinder/).

*Surveyor screening for GRMD editing gRNAs: No software was used.
*Design of single gRNA for WCMD and LRMD mutations: ESEfinder 3.0 software (Version 3.0, Cold Spring Harbor Laboratory, http://rulai.cshl.edu), uCRISPR algorithm (Zhang, D., Hurst, T., Duan, D. & Chen, S.J. Unified energetics analysis unravels SpCas9 cleavage activity for optimal gRNA design. Proc Natl Acad Sci U S A 116, 8693-8698, 2019), Cas-OFFfinder software (Version (no data), http://www.rgenome.net/cas-offinder/), and UCSC Genome Browser (Version (no data), https://genome.ucsc.edu).
*In vitro screening of the sgRNA for LRMD and WCMD editing: Image Studio™ Lite Software (Version 5.2.5, LI-COR Inc.)
*The gRNA cis-plasmids for the GRMD editing: No software was used.
*The gRNA cis-plasmids for the LRMD and WCMD editing: No software was used.
*AAV production and purification: No software was used.
*Transient immune suppression: No software was used.
*AAV administration: No software was used.
*Morphological analysis: ImageJ software (Version 1.48b, https://imagej.nih.gov)
*Western blot: Image Studio™ Lite Software (Version 5.2.5, LI-COR Inc.)
*Vector genome copy number quantification: Sequence Detection System (SDS) software (Version 2.4, ThermoFisher Scientific).
*Vector transcript quantification: Sequence Detection System (SDS) software (Version 2.4, ThermoFisher Scientific).
*Cytokine transcript quantification: QuantaSoft software (Version 1.0, Bio-Rad).
*Anti-SpCas9 antibody assay: Gen5 software (Version 3.04, BioTeck).
*SpCas9 enzyme linked immune absorbent spot (ELISpot) assay: No software was used.
*Serum Cytokine quantification: Belysa software (Version 1.0.19, MilliporeSigma).
*Blood chemistry: No software was used.
*Statistical analysis: GraphPad PRISM software version 8.0 (GraphPad Software Inc.), and Matlab software (version R2020a, Statistics and Machine Learning Toolbox 11.7).

For manuscripts utilizing custom algorithms or software that are central to the research but not yet described in published literature, software must be made available to editors and reviewers. We strongly encourage code deposition in a community repository (e.g. GitHub). See the Nature Portfolio guidelines for submitting code & software for further information.

Data

Policy information about availability of data

All manuscripts must include a data availability statement. This statement should provide the following information, where applicable:

- Accession codes, unique identifiers, or web links for publicly available datasets
- A description of any restrictions on data availability
- For clinical datasets or third party data, please ensure that the statement adheres to our policy

The data that support the findings of this study are available from the corresponding author upon reasonable request. All the raw data in this study are provided in the figures with the exception of the PCR gel electrophoresis, western blot, qPCR, ddPCR, and the anti-Cas9 antibody assay raw data. The unprocessed gels and blots are provided in supplementary figures 2, 3, 5, and 11. The source data for Figures 1c-i, l-p, r; 2b-e, g-j, l-p; 3d-g, k-n, p; and 4b-f, h-l, n-q; and supplementary figures 6a-e; 7b, g; 8b-c; 13 and 14 are provided with the paper. The vector genome copy number was determined by the cycle value from a quantitative TaqMan PCR reaction that was first converted to the total copy number in the reaction using a standard curve, and then divided by the amount of diploid genome present in the total ng of genomic DNA used in the reaction. The transcript copy number was determined by the cycle value from a qPCR reverse transcription (RT) reaction that was first converted to the raw copy number using a standard curve, and then divided by the total amount of cDNA (ng) used in the reaction. The cytokine transcript copy was determined by the droplet digital PCR (ddPCR) reaction and then divided by the total amount of cDNA (ng) used in the reaction. The anti-SpCas9 antibody concentration presented was determined by converting the reaction color absorbance value to ug/ml using a standard curve. The canFam3 assembly of UCSC Genome Browser (https://genome.ucsc.edu) was used to search for all the potential genome-wide off-target sites.

Field-specific reporting

Please select the one below that is the best fit for your research. If you are not sure, read the appropriate sections before making your selection.

[x] Life sciences [ ] Behavioural & social sciences [ ] Ecological, evolutionary & environmental sciences

For a reference copy of the document with all sections, see nature.com/documents/nr-reporting-summary-flat.pdf

Life sciences study design

All studies must disclose on these points even when the disclosure is negative.

Sample size	The sample size was determined based on the availability of experimental animals and AAV vectors. The sample size is summarized in Supp Table 7.
Data exclusions	No data was excluded.
Replication	For the vector genome copy number, transcript copy number, and Cas9-antibody measurements, technical replicates (n=2 to 3) were performed whenever samples were available. All attempts at replication were successful for these experiments. The Ct values of the standard curve samples were used to verify the reproducibility of the qPCR assay across independent qPCR runs. The number of droplets generated during the ddPCR assay was used to verify the reproducibility of the assay across all analyzed samples and independent ddPCR runs. A standard was used in each ELISA assay to assure the consistency of the assay.
Randomization	Randomization was not used in the study. Animals were allocated to research group based on the availability. Since all dogs had the same

	genetic background we didn't need to control for covariates.
Blinding	The Cas9-antibody, SpCas9 ELISpot, Cytokine transcript, and serum cytokine measurement was performed in a blinded fashion. No blinding was used in the other assays as they were performed by authors involved in sample collection from the treated animals.

Reporting for specific materials, systems and methods

We require information from authors about some types of materials, experimental systems and methods used in many studies. Here, indicate whether each material, system or method listed is relevant to your study. If you are not sure if a list item applies to your research, read the appropriate section before selecting a response.

Materials & experimental systems

n/a	Involved in the study	
☐	☒	Antibodies
☐	☒	Eukaryotic cell lines
☒	☐	Palaeontology and archaeology
☐	☒	Animals and other organisms
☒	☐	Human research participants
☒	☐	Clinical data
☒	☐	Dual use research of concern

Methods

n/a	Involved in the study	
☒	☐	ChIP-seq
☒	☐	Flow cytometry
☒	☐	MRI-based neuroimaging

Antibodies

Antibodies used

Dystrophin expression was detected using immunofluorescence staining with the following antibodies:
Primary antibody: Mouse monoclonal antibody against human dystrophin spectrin-like repeat 17 (Catalog#: MANEX44A, clone 5B2, 1:30, University of Iowa Hybridoma Bank, https://dshb.biology.uiowa.edu, a gift from Dr. Glen Morris).
Secondary antibody: Alexa Fluor 594 conjugated goat anti-mouse IgG(H+L) (A11020, Lot# 1946335, 1:100, ThermoFisher Scientific).

Flag-tagged SERCA2a was detected by immunofluorescence staining with the following antibody:
Primary antibody: Mouse monoclonal antibody against the Flag peptide (Catalog# F1804, Lot# SLBB7188, 1:500, MilliporeSigma, Burlington, MA, USA)
Secondary antibody: Alexa Fluor 594 conjugated goat anti-mouse IgG(H+L) antibody (A11020, Lot# 1946335, 1:100, ThermoFisher Scientific).

CD4+T Cell infiltration was detected using immunohistochemistry staining with the following antibodies:
Primary antibody: Rat monoclonal IgG2a antibody against canine CD4 [Catalog# MCA1038GA, clone YKIX 302.9, Lot # 1707, 1:1000, Bio-Rad (formerly AdD Serotec)].
Secondary antibody: Biotin conjugated goat anti-rat IgG (H+L) (A10517, Lot # 1441195, 1:1000, ThermoFisher Scientific).

CD8+T Cell infiltration was detected using immunohistochemistry staining with the following antibodies:
Primary antibody: Rat monoclonal IgG1 antibody against canine CD8 [Catalog# MCA1039, clone YCATE 55.9, Lot # 149349, 1:200, Bio-Rad (formerly AdD Serotec)].
Secondary antibody: Biotin conjugated goat anti-rat IgG (H+L) (A10517, Lot # 1441195, 1:1000, ThermoFisher Scientific).

MHCII was detected using immunohistochemistry staining with the following antibodies:
Primary antibody: Mouse monoclonal IgG1 antibody against canine MHC Class II Monomorphic [Catalog# MCA2037S, clone CA2.1C12, Lot # 151735, 1:100, Bio-Rad (formerly AdD Serotec)].
Secondary antibody: Biotin conjugated goat anti-mouse IgG1 (A10519, Lot # 2115665, 1:1000, ThermoFisher Scientific).

GranzmeB was detected using immunohistochemistry staining with the following antibodies:
Primary antibody: Rabbit polyclonal antibody against human GranzymeB (Catalog# E2580, Lot # GR3313356-1, 1:50, Spring Bioscience).
Secondary antibody: Biotin conjugated goat anti-rabbit polyclonal (B8895, Lot # 086M4770V, 1:500, MilliporeSigma).

FoxP3 was detected using immunohistochemistry staining with the following antibodies:
Primary antibody: Rat monoclonal IgG2a antibody against mouse FoxP3 [14-5773, clone FJK-16s, Lot # 2023701, 1:100, Thermo Fisher Scientific (formerly eBioscience)].
Secondary antibody: Biotin conjugated goat anti-rat IgG (H+L) (A10517, Lot # 1441195, 1:1000, ThermoFisher Scientific).

Dystrophin was detected by Western blot in a PDVF membrane using the following antibodies:
Primary antibody: Rabbit polyclonal antibody against the C-terminal domain of the dystrophin protein (Catalog RB-9024-P0, Lot# 9013p-1610E, 1:250, ThermoFisher Scientific).
Secondary antibody: Peroxidase conjugated goat anti-rabbit IgG antibody (AP132P, Lot# 3123491, 1:10,000, MilliporeSigma).

Cas9 was detected by Western blot in a PDVF membrane using the following antibodies:
Primary antibody: Rabbit monoclonal antibody against HA tag (Catalog 3724S, Lot# 9, 1:500, Cell Signaling Technology, Inc.)

Secondary antibody: Peroxidase conjugated goat anti-rabbit IgG antibody (AP132P, Lot# 3123491, 1:10,000, MilliporeSigma).

Vinculin was detected by Western blot in a PDVF membrane using the following antibodies:
Primary antibody: Mouse IgG1 monocolonal antibody (V9131, clone hVIN-1, ascites fluid, Lot # 036M4797V, 1:1000, MilliporeSigma).
Secondary antibody: Peroxidase conjugated goat anti-mouse IgG (H+L) antibody (AP124P, lot: 3032923, 1:10,000, MilliporeSigma).

Validation	All antibodies have been validated by the manufactures. Additional validations were performed in house as following. For immunostaining and immunohistochemical staining, validation was performed in the presence and/or absence of primary and/or secondary antibody. For western blot, the corresponding proteins were assessed using know positive controls as well as the protein size in the gel. Detailed validation of the dystrophin antibody is available at website https://journals.plos.org/plosone/article?id=10.1371/journal.pone.0088280

Eukaryotic cell lines

Policy information about cell lines

Cell line source(s)	Canine primary myoblasts were extracted in house according to a published protocol (Berg, Z., Beffa, L.R., Cook, D.P. & Cornelison, D.D. Muscle satellite cells from GRMD dystrophic dogs are not phenotypically distinguishable from wild type satellite cells in ex vivo culture. Neuromuscul Disord 21, 282-290, 2011). 293 cells used in AAV production were originally purchased from the ATCC (https://www.atcc.org/products/all/crl-3216.aspx) and were subsequently propagated in house.
Authentication	Authentication was based on the expected morphology of the cells.
Mycoplasma contamination	All cell lines were negative for mycoplasma contamination.
Commonly misidentified lines (See ICLAC register)	None commonly misidentified cell lines were used in the study.

Animals and other organisms

Policy information about studies involving animals; ARRIVE guidelines recommended for reporting animal research

Laboratory animals	All experimental dogs were generated at the University of Missouri by artificial insemination. They are on a mixed genetic background of golden retriever, Labrador retriever, beagle and Welsh corgi. All affected dogs carry null mutations in the dystrophin gene. All experimental dogs were housed in an AALAC accredited, limited access, conventional animal care facility and kept under a 12-hour light/12-hour dark cycle. Affected dogs were housed in a raised platform kennel while normal dogs were housed in a regular floor kennel. Depending on the age and size, two or more dogs are housed together to promote socialization. Normal dogs were fed dry Purina Lab Diet 5006 while affected dogs were fed wet Purina Proplan Puppy food. Dogs were given ad libitum access to clean drinking water. Toys were allowed in the kennel with dogs for enrichment. Dogs were monitored daily by the caregivers for overall health condition and activity. A full physical examination was performed by the veterinarian from the Office of Animal Research at the University of Missouri for any unusual changes in behavior, activity, food and water consumption, or when clinical symptoms were noticed. The body weights of the dogs were measured periodically to monitor growth. The age, sex, and sample size are summarized in Supplementary Table 7.
Wild animals	No wild animals were used.
Field-collected samples	No field-collected samples were used in the study.
Ethics oversight	All animal experiments that were performed at the University of Missouri were approved by the Animal Care and Use Committees of the University of Missouri. Serum from a subset of dogs shown in Supplementary Figure 6b were collected at Auburn University. This was approved by the Animal Care and Use Committee of Auburn University.

Note that full information on the approval of the study protocol must also be provided in the manuscript.
